# SETD2 loss in renal epithelial cells drives epithelial‐to‐mesenchymal transition in a TGF‐β‐independent manner

**DOI:** 10.1002/1878-0261.13487

**Published:** 2023-07-17

**Authors:** Tianchu Wang, Ryan T. Wagner, Ryan A. Hlady, Xiaoyu Pan, Xia Zhao, Sungho Kim, Liguo Wang, Jeong‐Heon Lee, Huijun Luo, Erik P. Castle, Douglas F. Lake, Thai H. Ho, Keith D. Robertson

**Affiliations:** ^1^ Molecular Pharmacology and Experimental Therapeutics Graduate Program, Mayo Clinic Graduate School of Biomedical Sciences Mayo Clinic Rochester MN USA; ^2^ Department of Molecular Pharmacology and Experimental Therapeutics Mayo Clinic Rochester MN USA; ^3^ Division of Biomedical Statistics and Informatics, Department of Health Science Research Mayo Clinic Rochester MN USA; ^4^ Epigenomics Development Laboratory Mayo Clinic Rochester MN USA; ^5^ Department of Laboratory Medicine and Pathology Mayo Clinic Rochester MN USA; ^6^ Division of Hematology and Oncology Mayo Clinic Arizona Phoenix AZ USA; ^7^ Department of Urology Tulane University New Orleans LA USA; ^8^ School of Life Sciences Arizona State University Tempe AZ USA

**Keywords:** clear cell renal cell carcinoma, epithelial‐to‐mesenchymal transition, histone H3 lysine 36 trimethylation, paracrine signaling, SETD2 mutation, transcription factors

## Abstract

Histone‐lysine *N*‐methyltransferase SETD2 (SETD2), the sole histone methyltransferase that catalyzes trimethylation of lysine 36 on histone H3 (H3K36me3), is often mutated in clear cell renal cell carcinoma (ccRCC). SETD2 mutation and/or loss of H3K36me3 is linked to metastasis and poor outcome in ccRCC patients. Epithelial‐to‐mesenchymal transition (EMT) is a major pathway that drives invasion and metastasis in various cancer types. Here, using novel kidney epithelial cell lines isogenic for *SETD2*, we discovered that SETD2 inactivation drives EMT and promotes migration, invasion, and stemness in a transforming growth factor‐beta‐independent manner. This newly identified EMT program is triggered in part through secreted factors, including cytokines and growth factors, and through transcriptional reprogramming. RNA‐seq and assay for transposase‐accessible chromatin sequencing uncovered key transcription factors upregulated upon *SETD2* loss, including SOX2, POU2F2 (OCT2), and PRRX1, that could individually drive EMT and stemness phenotypes in *SETD2* wild‐type (WT) cells. Public expression data from *SETD2* WT/mutant ccRCC support the EMT transcriptional signatures derived from cell line models. In summary, our studies reveal that SETD2 is a key regulator of EMT phenotypes through cell‐intrinsic and cell‐extrinsic mechanisms that help explain the association between SETD2 loss and ccRCC metastasis.

## Graphical abstract

Histone methyltransferase SETD2 catalyzes H3K36me3 at gene bodies and when mutated is associated with poor outcome in clear cell renal cell cancer. SETD2 loss results in activation of epithelial‐to‐mesenchymal transition (EMT), global H3K36me3 loss, and chromatin opening. The EMT and stemness phenotypes are driven intrinsically by increased transcription factor binding and extrinsically by paracrine effects mediated through secreted factors.

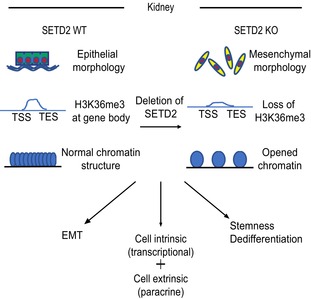

**Abbreviations**
ATAC‐seq: assay for transposase‐accessible chromatin sequencing
ccRCC: clear cell renal cell carcinoma
ChIP‐seq: chromatin immunoprecipitation sequencing
CM: conditioned media
EGFP: enhanced green fluorescent protein
EMT: epithelial‐to‐mesenchymal transition
FBS: fetal bovine serum
GSEA: gene set enrichment analysis
H3K36me3: histone H3 lysine 36 trimethylation
IFN: interferon
ITH: intratumoral heterogeneity
KO: knockout
PBRM1: polybromo 1
PDX: patient‐derived xenografts
RPTEC: renal proximal tubule epithelial cells
RT-qPCR: reverse transcriptase-quantitative polymerase chain reaction
TCGA KIRC: the cancer genome atlas kidney renal clear cell carcinoma collection
TF: transcription factor
TGF‐β: transforming growth factor‐beta
TME: tumor microenvironment
VHL: von Hippel–Lindau (gene)
WT: wild‐type

## 1 Introduction

Renal cancer is the eighth leading cause of cancer death in the US. Clear cell renal cell carcinoma (ccRCC) is the major subtype of renal cancer and accounts for the majority of deaths from kidney cancer. After loss of the von Hippel–Lindau (VHL) gene, which is insufficient to drive ccRCC on its own [1], the genetic landscape of ccRCC is dominated by mutations in epigenetic regulators SETD2, polybromo 1 (PBRM1), and BAP1. SETD2 mutation and/or loss of the mark it writes, histone H3 lysine 36 trimethylation (H3K36me3), is linked to poor outcome and metastasis in ccRCC [2, 3]. Indeed, mutations in SETD2 occur in ~ 25% of primary ccRCC but increase to > 60% in ccRCC metastases [4, 5, 6], suggesting SETD2 plays an important role in the transition from primary to metastatic ccRCC. Furthermore, an immunohistochemistry‐based study showed that nearly 50% of primary ccRCC lose H3K36me3 [7]. Distinct subclonal SETD2 mutations occur in spatially separate regions of ccRCC, indicating selection for SETD2 inactivation, yet the advantage this mutation confers to cancer cells and/or the tumor microenvironment (TME) remains unclear [8]. SETD2 loss is also linked to metastasis in pancreatic, lung, and prostate cancer [9, 10, 11, 12]. Thus, H3K36me3 deregulation drives oncogenesis and metastasis in cancers beyond RCC, suggesting that SETD2's tumor/metastasis suppressor function is conserved among different tissues and independent of mutations commonly linked to ccRCC like VHL and PBRM1 [10, 12].

The epithelial‐to‐mesenchymal transition (EMT) is a key process during tumor progression in which epithelial cells lose polarity and adhesion and gain the ability to be migratory. In cancer, EMT, driven by diverse oncogenic signaling pathways including transforming growth factor‐beta (TGF‐β), and Notch [13], is implicated in cell invasion, metastasis, tumor stemness, plasticity, and drug resistance. TGF‐β is a potent inducer of EMT through small/mothers against decapentaplegic phosphorylation and activation of a variety of downstream effector EMT transcription factors (TFs) [14, 15, 16, 17]. EMT is also a dynamic biological process, with cells existing in a continuum of meta‐stable, intermediate states between fully epithelial and fully mesenchymal phenotypes (termed hybrid or partial‐EMT states). EMT dynamics are driven both by cell intrinsic alterations (genetic and epigenetic factors) and cell extrinsic mechanisms (paracrine signaling) among cancer cells with heterogeneous mutation profiles and their TME [18, 19, 20]. Tumor cells subvert normal EMT programs, characterized by loss of epithelial markers (e.g., E‐cad, claudins, and occludins) and acquisition of a mesenchymal markers (e.g., vimentin, N‐cad, fibronectin, and α‐SMA). These molecular changes drive increased cell invasion through degradation of basement membranes, intravasation into circulation, and distant dissemination, resulting in metastases, which represent the primary cause of cancer‐related death [20].

SETD2 is a histone methyltransferase that specifically catalyzes H3K36me3. H3K36me3 is enriched in gene bodies and correlates positively with transcriptional activity [21]. This epigenetic mark has a well‐documented role in directing DNA methylation [22, 23], and its loss is linked to defective homologous recombination [24], deregulated gene expression [3], spurious transcription initiation [25], and aberrant RNA splicing [3]. Here, we demonstrate that SETD2 deletion in nontumorigenic renal epithelial cells enhances cell migration, invasion, and stemness through a TGF‐β‐independent EMT program. The SETD2‐induced expression and phenotypic changes were mediated in part by global chromatin structure alterations driving a distinct transcriptional program and by paracrine signaling mediated through secreted factors. In summary, we show that loss of SETD2 induces marked changes in cellular phenotype independent of other ccRCC mutations and consistent with the greater metastatic propensity of SETD2‐mutant ccRCC.

## 2 Materials and methods

### 2.1 Cell culture

Renal proximal tubule epithelial cells (RPTEC) (RRID:CVCL_K278) are cultured in DMEM:F12 media supplemented with 10% fetal bovine serum (FBS), minimal essential media nonessential amino acids (Thermo Fisher, Waltham, MA, USA; catalog #11140050), sodium pyruvate (Thermo Fisher; catalog #11360070), RPTEC growth kit (ATCC, Manassas, VA, USA; catalog #ACS‐4007) supplement A 5 mL, supplement B 8 mL, and 250 μg·mL^−1^ G418 (Thermo Fisher; catalog #10131035) for maintaining selection of hTERT expression. RCJ‐41T1 cells are derived from a PDX model as described [26] and cultured in DMEM:F12 media supplemented with 10% FBS. 786‐O cells (RRID:CVCL_1051) are cultured in RPMI 1640 media (Corning, Corning, NY, USA; catalog #10‐040‐CV) supplemented with 10% FBS. For calculation of doubling times, each cell line is seeded at 1 × 10^4^ cells/well in a 6‐well plate in normal complete media or 2% reduced serum media. Plates are placed in an IncuCyte system and monitored for growth at 10× magnification under phase contrast. Proliferation is quantified using the incucyte Base Analysis Software AI Confluence algorithm (Sartorius, Ann Arbor, MI, USA). Each data point is normalized by dividing confluency percent data by the day zero confluency percent. The human CellCheck 9 Plus panel (IDEXX BioAnalytics, Columbia, MO, USA) was used to authenticate all cell lines (performed roughly every 12 months of use). The CellCheck 9 Plus panel focuses on 9 genetic STR markers unique to human cell lines, as well as 10 microbial contamination tests. The panel compared the obtained genetic data against a comprehensive reference database containing known human cell lines and generated an identity matching score for each cell line. The matching scores for all our cell lines was 100% and aligned with the expected cell line of origin, confirming the cell line's authenticity. No positive microbial contamination was detected in any cultures, including mycoplasma.

### 2.2 Generation of CRISPR KO clones

RPTEC and RCJ‐41T1 SETD2 knockout (KO) clones were generated by inducing error prone repair in exon 3 of the SETD2 locus. The sgRNA sequence (TAGAATATGATGACCCTCGT) used for targeting exon 3 was cloned into the PX458 CRISPR/Cas9 targeting vector (Addgene, Watertown, MA, USA; #48138), which also contains an enhanced green fluorescent protein (EGFP) reporter (Fig. S1B). 10^6^ cells are nucleofected with this plasmid using SE buffer and pulse code EN‐138. Forty‐eight hours following nucleofection, the GFP+ cells were sorted and recovered in 100‐mm culture plates. Individual clones were then isolated under a microscope and transferred to a 96‐well plate for further expansion. Once confluent, individual clones were passaged 1 : 5 to individual wells of a 12‐well plate for continued expansion, while the remaining cells were lysed and collected for genomic DNA. Successfully targeted clones were determined by INDEL screening following polymerase chain reaction amplification and Sanger sequencing of exon 3. Mutational profiles of each clone were determined by deconvolution of the Sanger sequencing traces (Figs S1B and S5A) using Synthego's ICE analysis tool. Individual clones containing frameshift mutations were then functionally validated for SETD2 loss by western blot analysis of global H3K36me3 levels (Fig. 3A; Fig. S5B).

### 2.3 Lentiviral transduction

For generating SETD2 rescue RPTEC cell lines, 200 000 KO cells are seeded in a 100‐mm dish and transduced with lentiviral vector containing full‐length human SETD2 (tagged with 3× FLAG). After 48 h transduction, cells are placed under blasticidin (5 μg·mL^−1^) selection. Single colonies are picked after 10 days. Validation of positive SETD2 rescue clones is performed by western blotting for H3K36me3. For generating stable cell lines ectopically expressing SOX2, OCT2, and PRRX1, WT RPTEC cells are transduced with lentiviral vectors containing eGFP (control vector; Genecopoeia, Rockville, MD, USA; EX‐EGFP‐Lv181), SOX2 (Genecopoeia; EX‐T2547‐Lv181), OCT2 (Genecopoeia; EX‐A2204‐Lv181), and PRRX1 (Genecopoeia; EX‐T1345‐Lv181) for 48 h. Cells are then placed under puromycin selection (1.5 μg·mL^−1^) for selecting positive clones ectopically expressing each of the three genes. Whole cell lysate of bulk selected cells was prepared, and validation of expression of the three genes performed by western blot using anti‐FLAG antibody (Sigma‐Aldrich, St. Louis, MO, USA; Cat #F1804).

### 2.4 Reverse transcriptase‐quantitative polymerase chain reaction (RT‐qPCR), western blotting, and immunofluorescence

RNA is extracted using standard Trizol methods and used for RT‐qPCR reactions as described previously [27]. CDNA is synthesized using 2 μg of RNA and the high‐capacity cDNA reverse transcription kit (Thermo Fisher; catalog #4368814). Average *C*_t_ values of housekeeping genes RPL30, B2M, and RPL13A are used as an internal control. Samples are run in duplicate, and a one‐way ANOVA is used for statistical analysis. The primer sequences are shown in Table S1. Western blotting is performed as described in [27]. For whole cell extract, cell pellets are lysed in RIPA buffer. For subcellular fractionation, cells are first lysed in hypotonic lysis buffer followed by RIPA buffer and HCl for nuclear and histone extraction, respectively. For whole cell lysate and nuclear lysate, 60 μg of protein is loaded per lane and imaged using a Li‐COR system. Immunofluorescence microscopy for EMT gene expression was performed as in Ref. [27]. In brief, cells were fixed in paraformaldehyde, permeabilized with triton X‐100 for staining, then counterstained with 4′,6‐diamidino‐2‐phenylindole and mounted for analysis.

### 2.5 Antibodies and reagents

Primary antibodies used are CDH1 (Cell Signaling Technology, Beverly, MA, USA; #3195S), MUC1 (Abcam, Boston, MA, USA; #ab109185), SNAI2 (Cell Signaling Technology; #9585S), MMP2 (Proteintech, Rosemont, IL, USA; #10373‐2‐AP), CD44 (GeneTex, Irvine, CA, USA; GTX102111), CD34 (Proteintech; 60180), vimentin (Santa Cruz Biotechnology; B0719), total SMAD2 (Cell Signaling Technology; #3103S), phosphorylated SMAD2 (Cell Signaling Technology; #3108S), TGF‐β (Cell Signaling Technology; #3711S), H3K36me1 (Abcam; #ab9048), H3K36me2 (Cell Signaling Technology; #2901S), H3K36me3 (Active Motif; #61101), histone H3 (Abcam; #ab1791), SOX2 (R&D Systems; #AF2018‐SP), OCT2 (Thermo Fisher; #39‐5400), PRRX1 (Novus Biologicals, St. Charles, MO, USA; #NBP1‐06067), anti‐FLAG (Sigma‐Aldrich; #F1804), GAPDH (Cell Signaling Technology; #2118S), and lamin B1 (Proteintech; #12987‐1‐AP). Secondary antibodies are anti‐rabbit (Invitrogen, Waltham, MA, USA; #SA5‐3557) and anti‐mouse (LI‐COR, Lincoln, NE, USA; #926‐68070). Lentiviral vectors are eGFP (control vector; Genecopoeia; EX‐EGFP‐Lv181), SOX2 (Genecopoeia; EX‐T2547‐Lv181), OCT2 (Genecopoeia; EX‐A2204‐Lv181), and PRRX1 (Genecopoeia; EX‐T1345‐Lv181).

### 2.6 Conditioned media preparation

RPTEC cells (WT, SETD2 KO, and SETD2 KO rescue) are grown in 6‐well plates until 80% confluent and then washed with 1× PBS to completely remove FBS. Cells are starved and secreted factors are therefore released in the absence of serum by incubating them with serum‐free media for 24 h. After 24 h, the cell culture media is harvested, followed by centrifugation to remove any cells or cellular debris. The supernatant is then collected as conditioned media (CM) and used for subsequent experiments.

### 2.7 Wound healing, migration, transwell invasion, and 3D spheroid formation

#### 2.7.1 Migration

In brief, cells are grown to subconfluence and a scratch is made with a pipette tip in the middle part of the well to observe migration. Assays are performed in media with 2% serum that minimizes cell growth over the course of the assay. Each sample is run in duplicate. In addition, growth rates are calculated for each cell line as described previously; all SETD2 KO cells grow more slowly than their isogenic counterparts, further minimizing the impact of cell growth on the interpretation of scratch wound healing assays.

#### 2.7.2 Invasion

Cell invasion assays are performed as described [28]. Briefly, cells are starved for 24 h in serum‐free media and 4000 cells are seeded on 2× collagen IV‐coated inserts. Cells are then incubated at 37 °C for 24 h to observe invasion. Each sample is run in triplicate.

#### 2.7.3 Spheroid formation

The 3D spheroid formation assay was performed in 6‐well ultra‐low attachment plates with 50 000 cells seeded for each cell line.

### 2.8 RNA‐seq, ChIP‐seq, ATAC‐seq, and bioinformatic/statistical analysis

#### 2.8.1 Sample preparation

Three micrograms of RNA are used to create sequencing libraries. RNA‐seq is run on a NovaSeq 6000 with 150 bp PE reads using the TruSeq Stranded mRNA protocol. ChIP‐seq libraries are prepared as described previously [27, 29]. Briefly, 4 × 10^6^ cells are used for ChIP reactions for H3K36me3. 10^5^ cells are used for preparing assay for transposase‐accessible chromatin sequencing (ATAC‐seq) libraries based on a standard protocol. Libraries are sequenced on an Illumina HiSeq at the University of Minnesota Genomics Core Facility. Samples are run in triplicate for ATAC‐seq and duplicate for RNA‐seq and ChIP‐seq.

#### 2.8.2 Data analysis

RNA‐seq, ChIP‐seq, and ATAC‐seq analysis are performed as described [29, 30]. Sequencing quality metrics are summarized in Table S2. Transcript levels are quantified using salmon [31] and the *Homo sapiens* GRCh38 cDNA all fasta from Ensembl (ftp://ftp.ensembl.org/pub/release‐94/fasta/homo_sapiens/cdna/), then differential analysis is performed using deseq2 v1.26.0. Volcano plots are generated using ggplot2 v3.3.6. Pathway analysis is performed using gene set enrichment analysis (GSEA) from the Broad Institute (https://www.gsea‐msigdb.org/gsea/index.jsp) via the fgsea v1.14.0 r package using the Hallmark pathway set (h.all.v7.5.1.symbols), NABA‐secreted factors, and Ramalho stemness pathways. Both ATAC‐seq and ChIP‐seq are first aligned to the reference human genome using bwa v0.7.13 and bowtie2 v2.3.3.1, respectively. For H3K36me3 ChIP‐seq, all samples are normalized to a Drosophila spike‐in control (http://hgdownload.soe.ucsc.edu/goldenPath/dm6/bigZips/dm6.fa.gz) relative to human reads (http://hgdownload.soe.ucsc.edu/goldenPath/hg38/bigZips/hg38.fa.gz). Precompiled index files are available at: https://spiker.readthedocs.io/en/latest/bowtie2_index.html#bowtie2‐index, using the spiker pipeline (https://spiker.readthedocs.io/en/latest/installation.html). SETD2 WT and rescue reads are normalized using bamCoverage in deeptools 2.0. samtools v1.9 is used for generating bam files. Peaks are called using macs2 v2.2.7.1. Differential analysis of ATAC‐seq and ChIP‐seq are performed by diffbind v2.14.0. meme_suite (https://meme‐suite.org/) is used for identification of TF binding motifs. Specifically, using AME we identified EMT TF motifs that are enriched in SETD2 KO rescued ATAC‐seq peaks. Overall quality and sequencing depth metrics are summarized in Table S2. tobias v0.14.0 was used for TF footprinting [32]. ANOVA is used for calculating *P*‐values for pairwise comparisons among WT, SETD2 KO, SETD2 KO rescue, and TGF‐β‐treated/untreated WT conditions. Graphpad prism is used for all statistical analyses.

### 2.9 Data integration with TCGA‐KIRC

To identify SETD2 WT and mutant samples from TCGA, we queried TCGA‐KIRC (the cancer genome atlas kidney renal clear cell carcinoma collection) to identify samples with biallelic inactivation of SETD2 (3p loss + mutation). Due to the moderate frequency of H3K36me3 loss independent of SETD2 mutation as well as intratumoral heterogeneity (ITH), we further stratified these cases and selected the lowest expressing mutants (approximately 30% of available samples) and an equivalent number of high expressing SETD2 WT tumors. Tumor samples were age‐matched and all in the grade 2–3 range, resulting in 12 SETD2 wt/high expressors and 12 SETD2 mutant/low expressors for further analysis (Table S3). We then performed differential analysis of expression data of these 24 samples and compared this to the differential expression data derived from the RPTEC cell lines to further assess the relevance of the RPTEC model to primary tissue‐derived findings. The entire TCGA KIRC RNA‐seq dataset (606 samples) was used for comparing expression of the three candidate SETD2 effector genes (SOX2, OCT2, and PRRX1) between normal and tumor, or M0 (nonmetastatic) and M1 (metastatic) primary ccRCC. *P*‐values are calculated using the nonparametric Wilcoxon t‐test. Survival analyses were derived from the UCSC XenaBrowser using TCGA KIRC datasets.

## 3 Results

### 3.1 Establishment of a nontumorigenic epithelial cell line model for studying SETD2‐driven functions relevant to kidney cancer

**Fig. 1:**
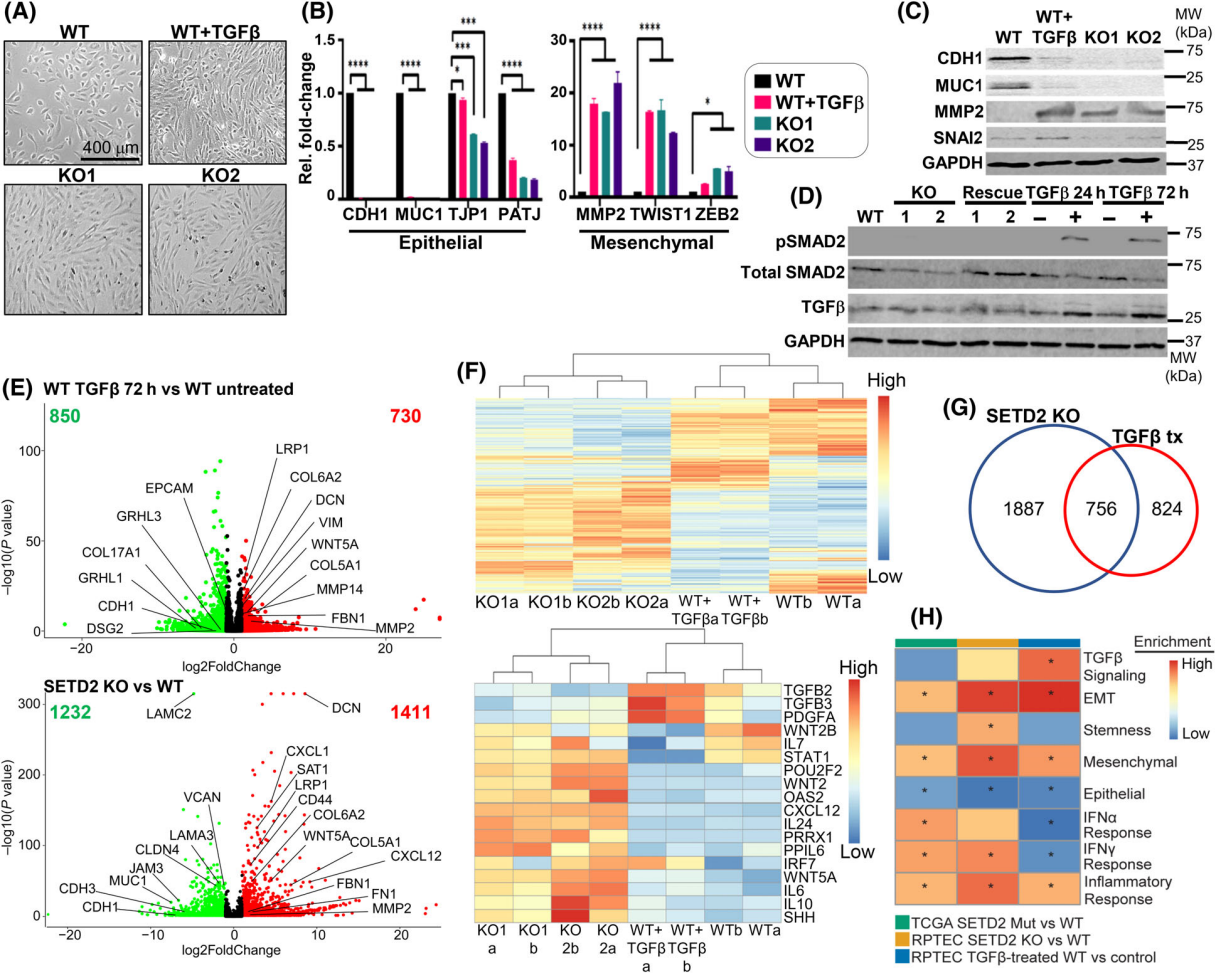
SETD2 inactivation induces a TGF‐β‐independent EMT program. (A) Cell morphology of WT, TGF‐β‐treated WT, and SETD2 KO RPTEC. Magnification 10×. Scale bar, 400 μm. (B) RT‐qPCR results for expression of epithelial and mesenchymal genes in WT, TGF‐β‐treated WT, and SETD2 KO clones. Data from three replicates are represented as mean ± SEM. *P*‐value is calculated for epithelial and mesenchymal genes individually using one‐way ANOVA. ****P < 0.0001; ***P < 0.001; *P < 0.05; ns, *P* ≥ 0.05. (C) Western blot showing expression of EMT markers in the indicated cell lines. (D) Western blot for total SMAD2 and phospho (Ser465/467) SMAD2, and TGF‐β levels in WT, SETD2 KO, SETD2 KO rescue, and TGF‐β‐treated/untreated WT RPTEC cells. Images in C and D are representative of three independent experiments. (E) Top panel: volcano plot of differentially expressed genes between TGF‐β‐treated WT and untreated control. Bottom panel: differentially expressed genes between SETD2 KO and WT RPTEC (genes shown are common between the two independent KO1/2 clones). Average value of log2‐fold change for each gene is used as the expression value. Green: downregulated genes (log2‐fold change < −1 and *P* < 0.05). Red: upregulated genes (log2‐fold change > 1 and *P* < 0.05). EMT genes are labeled. RNA‐seq was run in duplicate. (F) Heatmaps of all differentially expressed genes between SETD2‐deficient or TGF‐β treatment and RPTEC WT (top), and a subset of key genes linked to EMT, IFNγ, and secreted factors shown in the lower panel. ‘a/b’ denote replicates. (G) Venn diagram of differentially expressed genes between parental RPTEC, SETD2 KO RPTEC, and TGF‐β‐treated WT RPTEC. (H) Heatmap comparing enrichment of select GSEA hallmark pathways for differentially expressed genes between RPTEC WT/SETD2 KO (yellow), RPTEC TGF‐β‐treated vs control (blue), and TCGA primary SETD2 mutant vs WT tumors (green). *P*‐values are derived from the GSEA algorithm (Broad Institute); *P < 0.05. H3, histone H3; MW, molecular weight.

**Fig. 2:**
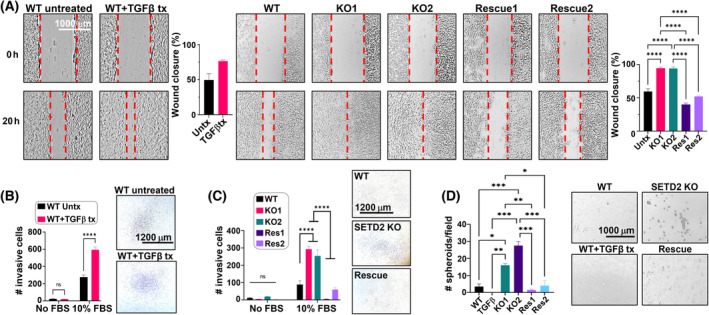
SETD2 loss drives EMT and stemness phenotypes. (A) Wound healing assay assessing migratory phenotype for 72 h TGF‐β treatment of RPTEC WT (left panel) vs untreated control and SETD2 KO vs WT/rescue (right panel). *P*‐value for comparing wound closure among WT, SETD2 KO, and SETD2 KO rescue RPTEC, each performed in duplicate, is calculated using one‐way ANOVA. Data are represented as mean ± SEM. Magnification 4×. Scale bar, 1000 μm. (B) Transwell assay testing invasiveness of TGF‐β‐treated WT cells. Magnification 2.5×. Scale bar, 1200 μm. (C) Transwell assay testing invasiveness of WT, SETD2 KO, and SETD2 KO rescue RPTEC. For B and C images of crystal violet‐stained cells that invaded through the membrane are shown beside the graphs. Two‐way ANOVA is used for statistical testing; samples are run in duplicate. Data are represented as mean ± SEM. Magnification 2.5×. Scale bar, 1200 μm. (D) 3D spheroid formation assay evaluating stemness in SETD2 KO cells. Data are represented as mean ± SEM. Image of spheroids in ultra‐low attachment plates are shown at the right. *P*‐value is calculated using one‐way ANOVA. Magnification 4×. Scale bar, 1000 μm. ****P < 0.0001; ***P < 0.001; **P < 0.01; *P < 0.05; ns, *P* ≥ 0.05.

Studying EMT in cancer cell lines that express known oncogenes (e.g., PTEN, KRAS) may confound the contribution of SETD2 to the process of tumorigenesis. Moreover, the presence of other driver mutations in ccRCC, such as VHL or PBRM1, often co‐mutated with SETD2, would complicate interpretation of SETD2‐specific contributions to EMT. To circumvent these issues, we used RPTEC immortalized with hTERT [33] as a surrogate for the normal renal epithelium. This cell type is also the putative cell of origin for ccRCC [34]. The overall experimental approach is summarized in Fig. S1A. Previous studies showed that RPTEC possess epithelial characteristics under normal growth conditions and undergo a physiologic EMT upon treatment with exogenous TGF‐β [35, 36]. When treated with 10 ng·mL^−1^ TGF‐β for 72 h, RPTEC display mesenchymal characteristics, including a change from cuboidal to spindle‐shaped morphology (Fig. 1A, top panel). Furthermore, RPTEC express epithelial genes (e.g., CDH1, MUC1) but not mesenchymal genes (e.g., MMP2, TWIST1, and SNAI2) as demonstrated by both RT‐qPCR and western blot (Fig. 1B,C). TGF‐β treatment of WT RPTEC also induced SMAD2 (Ser465/467) phosphorylation and stimulation of endogenous TGF‐β production, consistent with known mechanisms and downstream effectors of the TGF‐β pathway (Fig. 1D) [14, 15, 16, 17]. Global gene expression profiling by RNA‐seq confirms the differential expression of EMT‐related genes shown by RT‐qPCR and western blot and supports the observed induction of a mesenchymal phenotype in TGF‐β‐treated RPTEC (Fig. 1E, top panel). The changes in expression induced by TGF‐β are also summarized in heatmap form, with specific EMT‐related genes indicated (Fig. 1F top/bottom panels, Table S4). Consistent with gene expression changes, RPTEC treated with TGF‐β become more migratory in a wound healing assay (Fig. 2A, left panel) and more invasive in a transwell invasion assay (Fig. 2B). These data collectively indicate that RPTEC are responsive to TGF‐β and can serve as a model for studying the process of EMT independent of other genes commonly mutated in ccRCC.

**Fig. 3:**
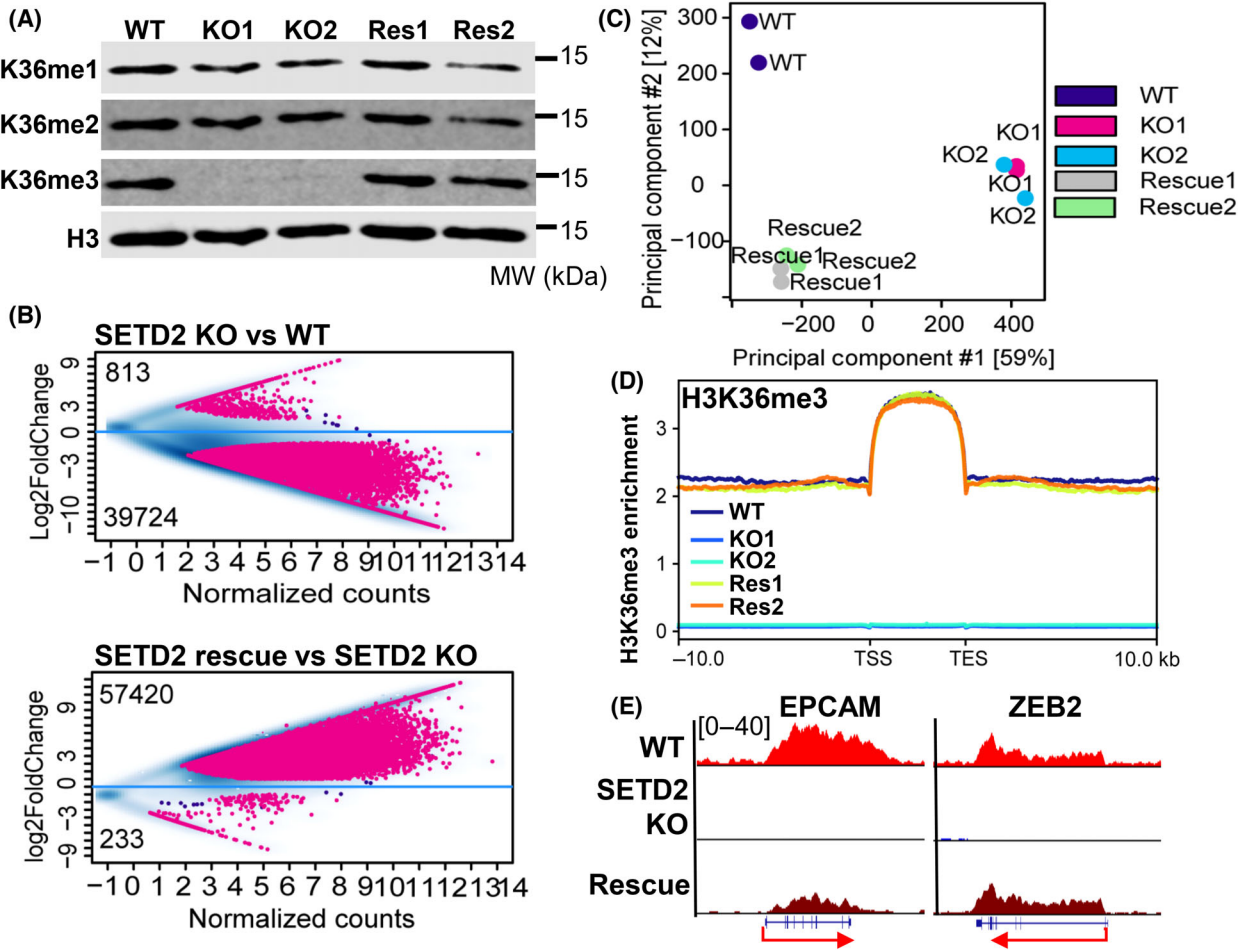
Global impact of SETD2 knockout and restoration on H3K36me3 distribution. (A) Western blot of H3K36me1, H3K36me2, and H3K36me3 levels in the indicated RPTEC lines. ‘Res’ is SETD2 KO rescue. Images are representative of three independent experiments. (B) MA plots of differential H3K36me3 peaks for the RPTEC SETD2 KO vs WT (top) and RPTEC SETD2 KO rescue vs KO (bottom) comparisons. ChIP‐seq was performed in duplicate for each cell line analysis. Significantly gained/lost peaks are highlighted in red (log2 fold change > 1, *P* < 0.05). (C) Principal component analysis of H3K36me3 ChIP‐seq data in RPTEC lines. Each sample was run twice as indicated by the colored circles. (D) Tag density plot of H3K36me3 enrichment for all protein‐coding genes at the gene body, 10 kb upstream of the TSS and 10 kb downstream of the TES. Data are derived from ChIP‐seq run in duplicate for each cell line. (E) Representative genome browser views of H3K36me3 levels at EMT‐related genes for the three isogenic RPTEC lines. TSS, transcription start site; TTS, transcription termination site.

To interrogate the impact of SETD2 loss on gene expression and EMT/metastasis‐related phenotypes in RPTEC, we used CRISPR/CAS9 with a guide RNA located in exon 3 of the *SETD2* gene to generate two independent KO clones for SETD2. Sanger sequencing confirms the presence of mutations that cause frameshifts on both SETD2 alleles in two clonally derived KO lines (referred to as KO1 and KO2, Fig. S1B). Consistent with the sequencing data, SETD2 KO RPTEC lines show near complete loss of H3K36me3 by western blot (Fig. 3A). The impact of SETD2 KO across the genome was further examined using ChIP‐seq for H3K36me3, which revealed marked loss of H3K36me3 peaks in SETD2 KO RPTEC (Fig. 3B, top panel). Principal component analysis shows marked segregation of the two KO clones from the WT line (Fig. 3C). A tag density plot and representative gene browser views for H3K36me3 demonstrate loss of overall and gene body enrichment of H3K36me3 (Fig. 3D,E). Taken together, these data demonstrate that we have established an isogenic SETD2 KO model in RPTEC and further reveal that SETD2 loss results in almost complete depletion of the H3K36me3 mark at the global level.

### 3.2 SETD2 inactivation induces a TGF‐β‐independent EMT program

Having established and validated a SETD2 KO model in RPTEC, we examined its impact on cellular phenotype. Morphologically, SETD2 KO RPTEC, like TGF‐β‐treated WT RPTEC, adopt a mesenchymal phenotype characterized by a spindle‐shaped appearance (Fig. 1A, bottom panel). Similarly, SETD2 inactivation downregulates epithelial markers and upregulates mesenchymal markers as shown by RT‐qPCR, western blotting, and RNA‐seq (Fig. 1B,C,E, bottom panel). Closer examination of differentially expressed genes reveals that SETD2 loss and TGF‐β treatment affect discrete and shared genes, with SETD2 showing a more pronounced expression signature (Fig. 1F,G). The most significantly enriched EMT‐related genes upon SETD2 loss/TGF‐β treatment is also summarized in heatmap form (Fig. 1F, bottom panel; Table S4). A Venn diagram summarizes the unique and overlapping effects of each condition (Fig. 1G). GSEA of TGF‐β‐regulated differentially expressed genes shows significant enrichment for the EMT pathway for the TGF‐β unique gene set (Fig. 1H). Interestingly, GSEA shows the EMT program is also enriched for the SETD2 unique gene set (Fig. 1H: 1887 genes in the Venn diagram, Fig. 1G). Furthermore, many genes enriched in the SETD2 loss‐driven EMT program are cytokines and chemokines including IL7, WNT2/5A, and IL6, suggesting that the SETD2 EMT program is induced, at least in part, by secreted factors (Fig. 1F, bottom, H). Unlike TGF‐β‐treated WT RPTEC, SETD2 KO cells also demonstrate activation of other unique pathways, including inflammatory and interferon responses. Moreover, compared to TGF‐β‐treated WT cells, the stemness‐related pathway is uniquely upregulated in SETD2 KO differentially expressed genes (Fig. 1H). Downregulation of representative epithelial genes (CDH1 and MUC1) and upregulation of representative mesenchymal genes (MMP2 and SNAI2) at the protein level was confirmed using immunofluorescence staining (Fig. S2A,B). Given the largely distinct set of genes regulated by SETD2, yet the common phenotypic link of EMT, we examined whether the SETD2 KO‐induced gene expression changes are mediated through TGF‐β. Consistent with a novel pathway, SETD2 KO cells did not show induction of TGF‐β, whereas exogenous treatment of WT cells with TGF‐β induced endogenous TGF‐β production as would be expected [14, 15, 16]. In addition, unlike TGF‐β‐treated WT RPTEC cells, SETD2 KO cells failed to show induction of SMAD2 phosphorylation, a key downstream effector for canonical TGF‐β signaling (Fig. 1D).

To investigate the extent to which our SETD2 isogenic model reflects SETD2 loss in primary human ccRCC tissue, we utilized the TCGA KIRC database for gene expression profiles. Given both the marked ITH known to occur in ccRCC [8] and the moderately frequent SETD2‐mutation‐independent loss of H3K36me3 and SETD2 downregulation [3, 37], we curated a ‘high confidence’ set of primary ccRCC with intact SETD2 (no SETD2 mutation with high expression) to compare with SETD2‐deficient ccRCC (biallelic SETD2 inactivation accompanied by reduced expression; 12 ccRCCs from each group (Table S3)). This is an especially important distinction as TCGA does not have H3K36me3 status available, so controlling for the expression status of the writer of H3K36me3 (SETD2) is essential for accurately comparing SETD2‐proficient to SETD2‐deficient tumors [37]. Using RNA‐seq data for these 24 samples, we performed principal component analysis in an unsupervised approach, revealing that SETD2 wild‐type and SETD2 mutant/low tumors largely segregate, and that 1702 genes are differentially expressed between these groups, indicating that SETD2 loss drives a distinct transcriptional signature in primary ccRCC as well (Fig. S3A,B). Indeed, comparing GSEA pathway analysis among SETD2 differentially expressed genes from TCGA‐KIRC, RPTEC SETD2 KO, and TGF‐β‐regulated genes discussed previously shows that while the TGF‐β signaling pathway is uniquely enriched in TGF‐β‐treated WT RPTEC, many other pathways, including EMT, inflammation, and interferon signaling, are enriched in both the SETD2‐mutant ccRCC samples from KIRC and the SETD2 KO RPTEC model (Fig. 1H). Moreover, using custom epithelial and mesenchymal gene signatures derived from published gene sets [13], we observe that a mesenchymal gene signature is enriched in SETD2 KO, TGF‐β‐treated WT, and SETD2‐mutant ccRCC samples, while an epithelial expression signature is depleted (Fig. 1H). Finally, we demonstrate that the differentially expressed gene set from TCGA SETD2 WT and mutant samples efficiently segregates the RPTEC WT from SETD2 KO isogenic cell lines (Fig. S3C). These data collectively show that SETD2 loss generates a distinctive transcriptional signature conserved between our cell line model and primary tissues, which is characterized by EMT and inflammatory expression profiles.

### 3.3 SETD2 loss promotes EMT and stemness phenotypes

We next assessed the effect of SETD2 inactivation on cellular phenotypes related to EMT. In both wound healing migration and transwell invasion assays, SETD2 KO RPTEC were more migratory and invasive than their isogenic SETD2 WT counterparts (Fig. 2A, right panel, C). This difference in migration and invasion was not attributable to SETD2 KO cells acquiring a faster growth rate; rather the SETD2 KO lines grow more slowly than their WT counterparts under both standard growth conditions and the low serum growth condition (2% serum) used for the migration assays (Fig. S4A, Table S5). Moreover, SETD2 KO cells migrated to a greater extent than TGF‐β‐treated WT cells. (Fig. 2A, compare left and right panels). Unlike WT RPTEC cells treated with TGF‐β, however, SETD2 KO cells readily formed spheroids on ultra‐low attachment cell culture plates, a property associated with cells undergoing EMT (Fig. 2D) [38]. This result is consistent with enrichment of stemness‐related pathways by GSEA (Fig. 1H) and confirmed by induction of a number stemness genes like CD44, associated with stemness and aggressive ccRCC (Fig. S2C) [39] in SETD2 KO cells, and those relevant to the early mesodermal lineage from which the kidney arises (e.g., SALL1, SIX2, and CITED1, Fig. S4B).

While tumor cell lines are not ideal for studying EMT, we nonetheless examined whether SETD2 inactivation would further shift cancer cells along the epithelial –mesenchymal spectrum. We used an existing isogenic SETD2 KO model in the 786‐O ccRCC cell line that we created previously [22] and a second ccRCC cell line derived from a treatment refractory Mayo Clinic ccRCC patient [26] (referred to as RCJ‐41T1) with SETD2 deficiency engineered using CRIPSR/CAS9 (similar to the RPTEC approach). Validation of the isogenic KO in both 786‐O and RCJ‐41T1 by Sanger sequencing (for RCJ‐41T1) and western blotting for H3K36me3 shows near complete loss of H3K36me3 in both SETD2 KO lines (Fig. S5A,B). We then tested these models in wound healing migration and transwell invasion assays. SETD2 KO cells were more migratory and invasive than their respective WT isogenic cells for both 786‐O and RCJ‐41T1 models (Fig. S5C,D), consistent with the phenotypic results in RPTEC. Like RPTEC, 786‐O and RCJ‐41T1 SETD2 KO cells grew similarly or more slowly than their SETD2 WT counterparts, which only further highlights the enhanced migration phenotype by excluding confounding effects of enhanced growth rate of SETD2 KO lines in migration assays (Table S5). Taken together, results from our isogenic SETD2 KO models reveal some similarities with canonical TGF‐β‐induced EMT, primarily at the phenotypic level, but also uncover many differences, including a distinct transcriptional signature, greater migratory capacity, and induction of stemness that appear to be independent of canonical TGF‐β signaling. These findings collectively suggest that loss of SETD2 induces EMT through a mechanism distinct from that of TGF‐β.

### 3.4 Paracrine signaling consequences of SETD2 inactivation

**Fig. 4:**
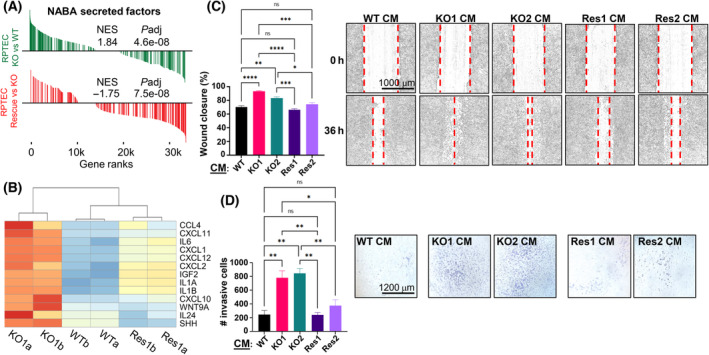
SETD2 loss drives an EMT program through paracrine signaling. (A) GSEA enrichment plots for the NABA‐secreted factors pathway showing enrichment in SETD2 KO vs WT RPTEC, with significant reversal in the SETD2 rescue vs KO comparison. Normalized enrichment scores (NES) and adjusted *P*‐values (*P*adj) are shown. (B) A corresponding heatmap of a subset of NABA‐secreted factors from (A) in replicates of WT, KO, and rescue RPTEC. ‘a/b’ indicate replicates. The color scale is the same as in Fig. 1. (C) A bar chart and representative examples of a wound healing assay assessing migration capacity of WT RPTEC cells treated with CM from RPTEC WT, SETD2 KO, and SETD2 KO rescue cells. Magnification, 4×. Scale bar, 1000 μm. (D) Summary bar chart and representative examples from a transwell assay testing invasiveness of WT cells exposed to the same CMs as in C from RPTEC WT, KO, and rescue cells. Magnification, 2.5×. Scale bar, 1200 μm. One‐way ANOVA is used for statistical testing in B and C. ****P < 0.0001; ***P < 0.001; **P < 0.01; *P < 0.05; ns, *P* ≥ 0.05. All reactions were performed in duplicate.

Our previous gene expression and pathway analyses revealed that SETD2 inactivation promoted a pro‐inflammatory expression phenotype along with induction of interferon and cytokine pathways (Fig. 1F,G). Since these findings suggested that a non‐cell autonomous mechanism is contributing to the migration and invasion phenotypes induced by SETD2 inactivation, we further examined our data for secreted paracrine signaling molecules and their receptors that might stimulate EMT in the RPTEC model. Analysis of genes uniquely altered by SETD2 loss with GSEA confirms the significant enrichment for secreted factors characterized by cytokines, chemokines, and growth factor‐related genes (Fig. 4A,B; Table S6). To determine if these secreted factors contribute to the previously described EMT phenotypes induced by SETD2 inactivation, we prepared CM (as described in the Methods) from both WT and SETD2 KO RPTEC lines. Upon incubation of WT RPTEC with each CM, the SETD2 KO CM induced significantly faster wound healing migration and greater cell invasion than CM from SETD2 WT cells (Fig. 4C,D). These findings indicate that cell–cell contact is not required and the effect of SETD2 loss in driving EMT‐related phenotypes is likely regulated by both cell autonomous transcriptional changes and non‐‐cell autonomous factors working through the TME.

### 3.5 SETD2 rescue partially restores global transcriptional patterns and reverses EMT and stemness phenotypes

To determine if the SETD2 transcriptional and EMT‐related phenotypes are reversible and identify effects most directly related to the action of SETD2 (and its mark H3K36me3) rather than indirect targets, we transduced RPTEC SETD2 KO cells with a lentiviral expression vector containing full‐length WT human SETD2 (3×FLAG‐tagged, Fig. S1A). Following selection and derivation of stable single‐cell clones, we identified two clones that showed global rescue of H3K36me3 levels comparable to that in WT cells by western blotting (Fig. 3A). To further characterize these SETD2 rescue RPTEC lines (referred to as Rescue1 and Rescue2), we performed ChIP‐seq for H3K36me3. The total number of H3K36me3 peaks was modestly higher in the rescue lines than in parental RPTEC, presumably due to the ectopic expression system (Fig. 3B, lower panel). Nonetheless, differential analysis reveals dramatic recovery of H3K36me3 peaks in rescue cells (Fig. 3B,D,E). Principal component analysis shows a marked shift of KO lines toward WT cells upon SETD2 rescue on PC1, which accounts for most of the variance (Fig. 3C). Furthermore, both tag density plots and representative browser views show that H3K36me3 returns to gene bodies at near WT levels for most loci (Fig. 3D,E).

**Fig. 5:**
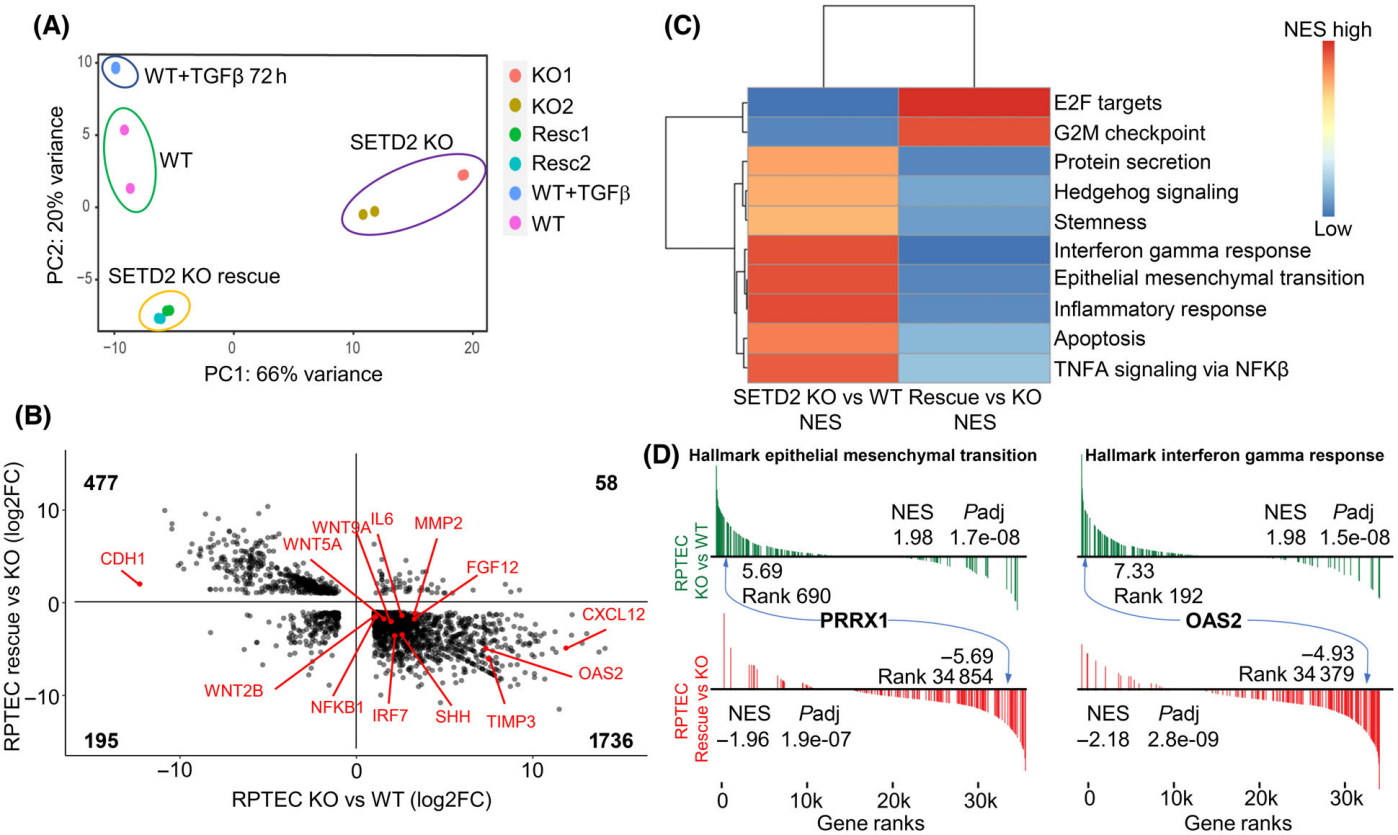
SETD2 rescue partially restores global transcriptional patterns and reverses EMT and stemness transcriptional signatures. (A) Principal component analysis of all genes in untreated RPTEC WT, SETD2 KO, SETD2 KO rescue, and TGF‐β‐treated WT RPTEC derived from RNA‐seq (all lines are run in duplicate). (B) A scatterplot of RPTEC KO vs WT against SETD2 rescue vs KO showing reversal of genes differentially expressed with SETD2 loss with ectopic re‐expression of SETD2. A subset of key EMT genes is labeled in red. (C) A heatmap of hallmark pathways from GSEA altered in SETD2 KO and reversed with ectopic re‐expression. (D) A subset of reversed pathways from (C) demonstrating reversal of EMT and IFNγ pathways with reintroduction of SETD2. Two genes of interest are indicated (PRRX1 and OAS2), and the differential expression and gene rank in their respective comparisons are shown, along with the pathway normalized enrichment score (NES) and adjusted *P*‐value (*P*adj).

Having shown that ectopic re‐expression of SETD2 rescues gene body loss of H3K36me3 in SETD2 KO cells, we examined the effect of SETD2 rescue on the transcriptome. Principal component analysis of RNA‐seq data, like the H3K36me3 PCA, shows that the global transcriptome is largely rescued upon ectopic SETD2 re‐expression, as indicated by the clustering of rescue cells closer to WT parental cells on PC1, which accounts for most of the variance (Fig. 5A). The effect of TGF‐β treatment of WT cells is also indicated for comparison and highlights the much more dramatic alteration to the transcriptome caused by SETD2 depletion compared to that of TGF‐β treatment. Further comparison of genes significantly changed in both conditions (that is, SETD2 KO vs WT and rescue vs SETD2 KO) demonstrates that nearly 85% of this gene set shows evidence of rescue at the transcriptional level (Fig. 5B). GSEA for the rescued gene set (genes overlapping between the SETD2 KO vs WT and rescue vs SETD2 KO comparisons) showed that the EMT program is largely reversed compared to the original SETD2 KO vs WT comparison (Fig. 5C,D). A fraction of genes significantly rescued in the RPTEC model overlaps with the SETD2‐regulated gene set derived from TCGA‐KIRC (Fig. S3C,D) and clusters the rescue RPTEC with the WT cells (Fig. S3C). The differentially expressed gene set derived from TCGA‐KIRC SETD2 wt/mutant tumors also shows evidence of reversal upon SETD2 rescue (Fig. S3D), further emphasizing that the patient‐derived SETD2 mutational signature is being replicated in the RPTEC model. We next examined EMT‐related phenotypes in the RPTEC SETD2 rescue clones. Wound healing and transwell invasion assays demonstrate significantly reduced migration and invasion for SETD2 rescue cells, with the rescue cells now behaving similarly to WT cells (Fig. 2A, right panel, C). In addition, the ability of SETD2 KO RPTEC to form spheroids was completely abrogated upon SETD2 rescue (Fig. 2D), and the effects of SETD2 KO CM on stimulating migration and invasion of WT cells via paracrine signaling were also abolished upon re‐expression of SETD2 (Fig. 4B–D). In summary, ectopic re‐expression of SETD2 rescues H3K36me3 levels both globally and regionally at gene bodies, partially restores the transcriptome, and completely reverses the EMT‐related invasion and stemness phenotypes acquired upon loss of SETD2 function.

### 3.6 Chromatin accessibility measures reveal that SETD2 loss leads to global chromatin opening and uncover novel putative transcriptional effectors

**Fig. 6:**
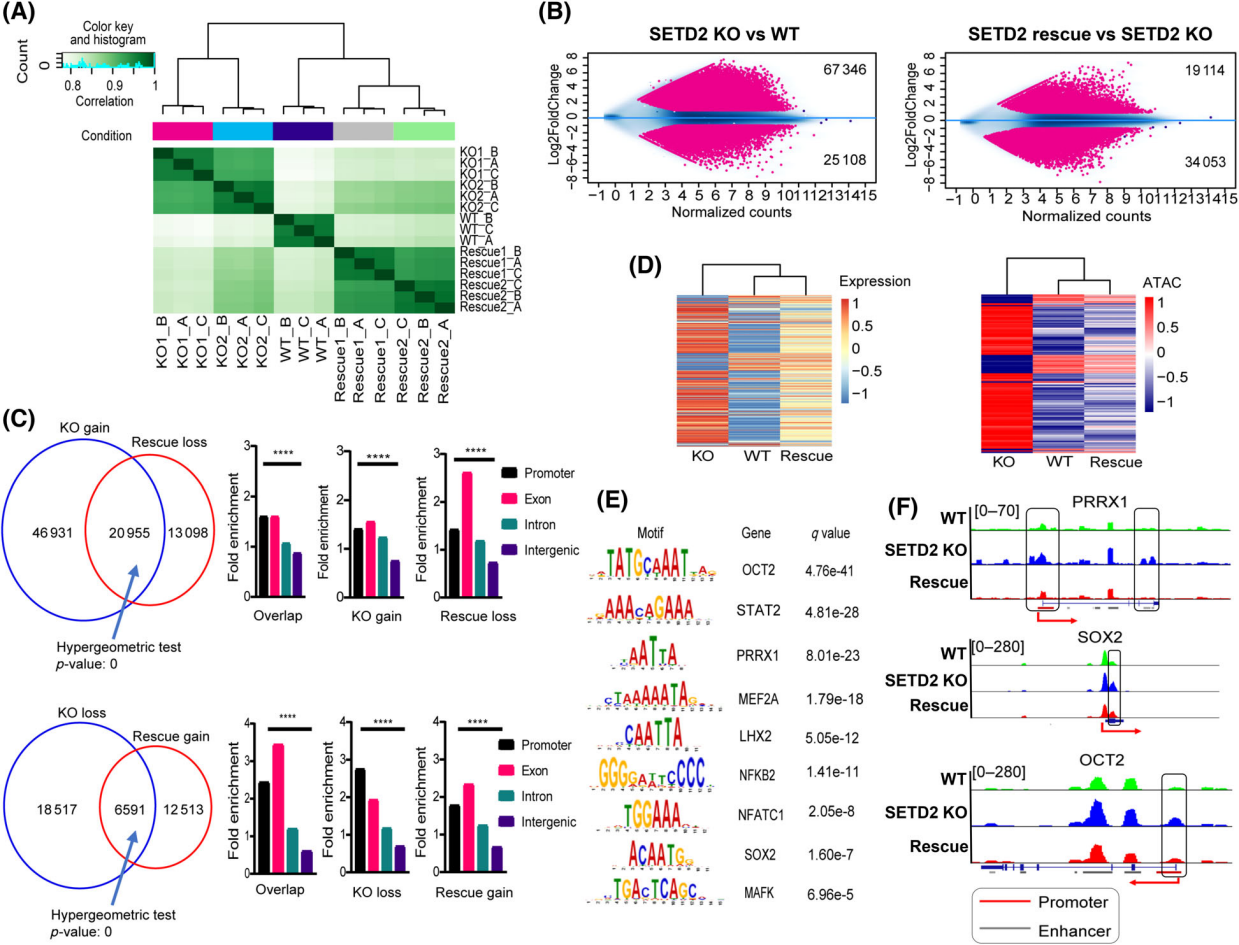
Global chromatin opening and novel transcriptional effectors of SETD2 loss revealed through ATAC‐seq. (A) Pairwise correlation of global ATAC‐seq data of WT, SETD2 KO, and SETD2 KO rescue RPTEC. Each cell line was run in triplicate for ATAC‐seq. (B) Differential analysis of ATAC‐seq peaks between RPTEC SETD2 KO vs WT, and SETD2 rescue vs KO as MA plots. Peaks with either log2‐fold change > 1 or < −1, *P* < 0.05 are highlighted in red. (C) Top panel: Venn diagram for overlapping ATAC‐seq peaks gained in SETD2 KO (67 346 peaks in B, left panel) and peaks lost in SETD2 KO rescue cells (34 053 peaks in B, right panel). Right panel: annotation of each of the three peak sets (gained, lost, and shared) to genomic features. Bottom panel: ATAC‐seq peaks lost in KO2 (25 108 peaks in B, left panel) and ATAC‐seq peaks gained in SETD2 KO rescue cells (19 114 peaks, right panel), and the distribution of these peaks in the genomic features indicated. *P*‐value for significance of enrichment of ATAC peaks within each feature relative to background is calculated using the Chi‐square test with Yates continuity correction. ****P < 0.0001. (D) Supervised clustering of genes whose expression is restored in SETD2 KO rescue cells and the correlation with corresponding reversed/rescued ATAC‐seq peaks. (E) TF motif analysis performed by AME in the MEME suite showing motifs significantly enriched in ATAC SETD2 KO rescued peaks in C (20 555 overlapped peaks in top panel and 6591 overlapped peaks in bottom panel). (F) Genome browser views of ATAC signals for representative EMT TFs in RPTEC WT, SETD2 KO, and rescue cell lines. Promoters and enhancers are indicated by red and gray lines, respectively. Regions of differential peaks are boxed.

To understand how H3K36me3 impacts chromatin structure and identify key transcriptional regulators involved in modulating the SETD2 EMT program, we assessed genome‐wide chromatin accessibility in RPTEC WT, SETD2 KO, and SETD2 KO rescue lines using ATAC‐seq. Pairwise correlation analysis shows globally that WT cells cluster with SETD2 rescue (albeit in different subclusters), and both further segregate from the SETD2 KO clones (Fig. 6A). Based on differential analysis, we observed more ATAC‐seq peaks gained in SETD2 KO relative to WT cells, whereas in SETD2 rescue cells more peaks are lost compared to KO, indicating that depletion of SETD2 induces global chromatin opening, which is partially reversed upon ectopic re‐expression of SETD2 (Fig. 6B). By comparing SETD2 KO and rescue differential ATAC‐seq peaks, then annotating them to genomic features, we observed that the chromatin accessibility differences are mostly located within promoters and exonic regions (Fig. 6C). Furthermore, by linking expression data of rescued genes to ATAC‐seq data of rescued peaks, we observed that overall gene expression correlates strongly with chromatin accessibility (Fig. 6D). Indeed, genes significantly upregulated in SETD2 KO and reversed in rescue have more accessible chromatin in SETD2 KO cells but less accessible chromatin in SETD2 rescue cells (Fig. 6D). Genome browser views of ATAC‐seq signal for representative EMT‐related genes are shown in Fig. S6A.

We next examined whether specific TFs are associated with significant alterations in chromatin accessibility that may provide candidate effectors of SETD2 loss on gene expression and EMT phenotypes. To do this, we focused on the most robust set of ATAC‐seq peaks that are reversed upon SETD2 rescue (Fig. 4C, overlapped peak sets in both Venn diagrams) since these are likely to be the most direct targets of SETD2 activity. TF motifs in these rescued ATAC‐seq peaks were identified using the MEME suite. We discovered many EMT/stemness‐related TF motifs significantly enriched for the rescued ATAC‐seq peak set (Table S7), including SOX2, PRRX1, and OCT2 [40, 41, 42], and TFs involved in driving inflammation and cytokine/chemokine secretion including STAT2 [43] and NFKB2 (Fig. 6E) [44, 45]. To further investigate the relevance of our TFs of interest (SOX2, OCT2, and PRRX1), we loaded our ATAC‐seq peaks into TOBIAS (Transcription factor Occupancy prediction By Investigation of ATAC‐seq Signal) [32] to uncover TF footprinting in SETD2 KO vs WT RPTEC cells. TOBIAS was designed to make use of ATAC‐seq data to predict TF binding and to correct for Tn5 transposase bias. To that end, we identified several dozen TFs with differential binding scores in mutant vs WT cells, where all three TFs of interest had higher differential binding scores in SETD2 KO vs WT cells, with PRRX1 and OCT2 being in the top 10% of all TFs with increased differential binding scores (Fig. S7A, Table S8). When the TF footprints for OCT2, PRRX1, and SOX2 are overlaid with their associated genes, we observe a general upregulation of this gene set using RNA‐seq from RPTEC SETD2 KO vs WT conditions, giving further credence to these TFs being relevant as effectors of SETD2‐mediated gene deregulation (Fig. S7B). Moreover, examination of the top 20 genes targeted by each TF demonstrate significant upregulation of these target genes, with multiple genes including those involved in canonical WNT signaling such as LGR5 (a shared target of SOX2 and OCT2), and PDGFRA (target of PRRX1) being linked to EMT (Fig. S7C). With the published epithelial and mesenchymal gene sets used previously in Fig. 1G that are in common with SETD2 KO RPTEC differentially expressed genes, we found that chromatin was overall less accessible at epithelial genes in SETD2 KO cells but more open at mesenchymal genes (Fig. S6B). Taken together, these data reveal that global changes in H3K36me3 lead to significant changes in chromatin structure that directly impact gene expression. Such changes are largely reversible with ectopic SETD2 re‐expression, and the focus on rescued ATAC‐seq peaks led to the discovery of SETD2‐regulated stemness/EMT/inflammation‐related TFs that may represent downstream effectors of the SETD2 loss‐driven EMT program.

### 3.7 SOX2, OCT2, and PRRX1 are downstream effectors of the SETD2‐regulated EMT program

**Fig. 7:**
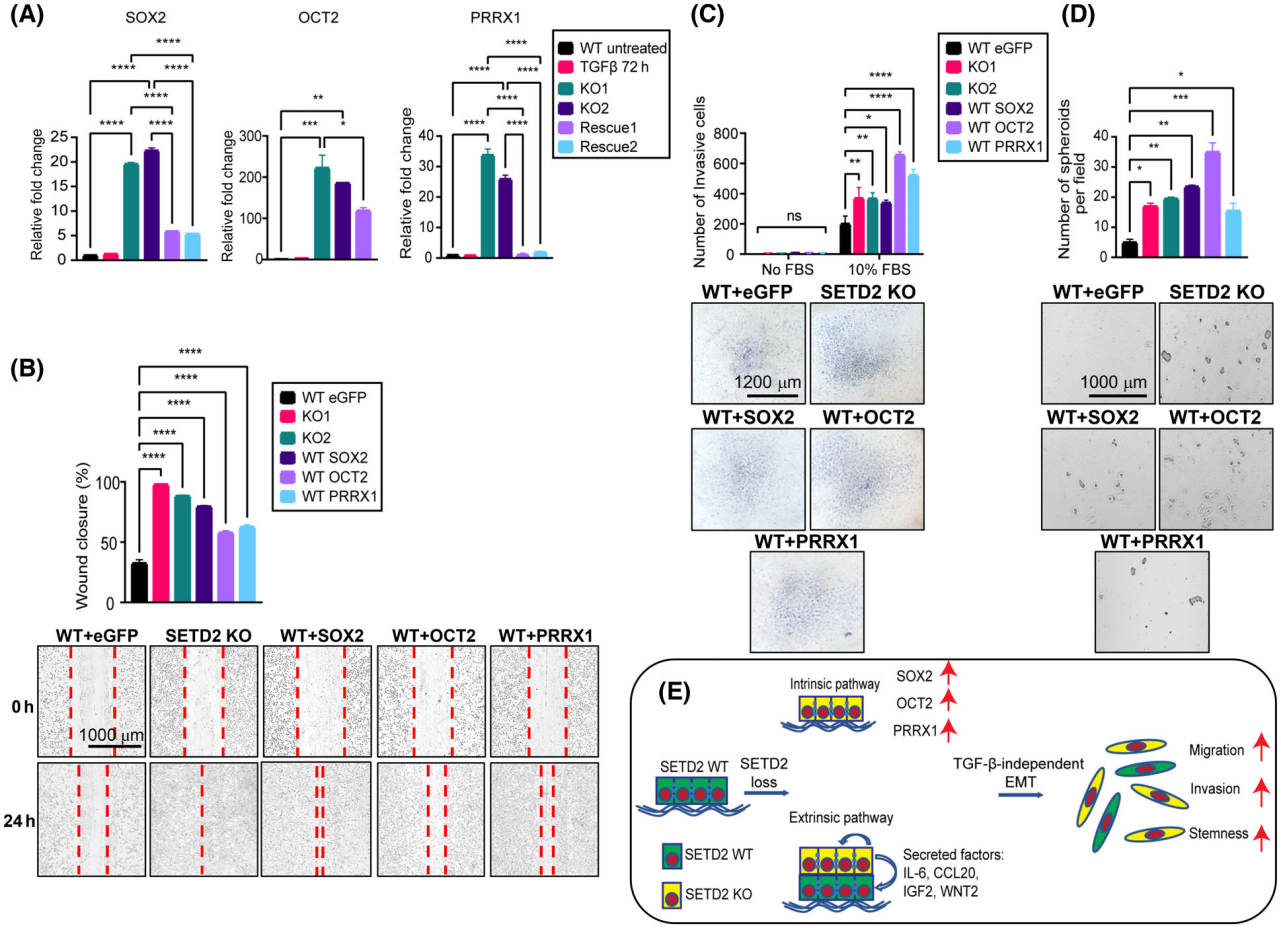
SOX2, OCT2, and PRRX1 are downstream effectors of the SETD2‐regulated EMT program. (A) Expression of SOX2, OCT2, and PRRX1 in TGF‐β‐treated WT (72 h), SETD2 KO, and SETD2 rescue tested by RT‐qPCR (run in triplicate). (B) Migration capacity by wound healing assay, (C) invasiveness by transwell assay, and (D) stemness by 3D spheroid formation assay in RPTEC WT GFP (control vector), SETD2 KO1 and KO2, and SOX2/OCT2/PRRX1‐transduced WT RPTEC lines. Images are taken at 4× magnification, scale bar: 1000 μm for (B) and (D) and at 2.5× magnification, scale bar: 1200 μm for (C). Data are represented as mean ± SEM for triplicate reactions for B–D. *P*‐value is calculated by one‐way ANOVA in (A), (B), and (D). Two‐way ANOVA is used for statistical test for (C). ****P < 0.0001; ***P < 0.001; **P < 0.01; *P < 0.05; ns, *P* ≥ 0.05. (E) Model of the SETD2 loss‐driven EMT program through cell intrinsic (transcriptional) and cell extrinsic (paracrine) mechanisms.

Given that SOX2, OCT2, and PRRX1 are associated with EMT, stemness, and metastasis [40, 41, 42], and DNA binding motifs for these transcriptional regulators are significantly enriched for SETD2 KO rescued ATAC‐seq peaks, we sought to functionally test whether they act as downstream effectors of the SETD2 loss‐driven EMT program. All three genes are substantially upregulated upon SETD2 KO and largely reversed in SETD2 rescue RPTEC (Fig. 7A; Fig. S8A), consistent with the changes in ATAC‐seq signals at these loci (Fig. 6F). Expression of SOX2, OCT2, and PRRX1 are not induced in TGF‐β‐treated WT cells, highlighting that their expression is unique to the SETD2 transcriptional program (Fig. 7A; Fig. S8A). To determine the extent to which these three genes contribute to SETD2 loss‐driven EMT and stemness phenotypes, we ectopically expressed each factor by lentiviral transduction in WT RPTEC (where they are essentially not expressed). After validating ectopic expression at the protein level by western blot (Fig. S8B), we performed functional assays. WT parental RPTEC transduced with SOX2, OCT2, or PRRX1 became more migratory in wound healing assays (Fig. 7B), more invasive in transwell invasion assays (Fig. 7C), and more stem‐like in the 3D spheroid formation assay (Fig. 7D) than cells transduced with an eGFP control vector. Thus, ectopic expression of each of these SETD2 target genes can largely recapitulate the EMT‐related phenotypes observed in SETD2 KO cells.

To extend the relevance of our cell line‐derived findings to primary ccRCC, we examined expression of SOX2, OCT2, and PRRX1 in TCGA's KIRC dataset. Compared to normal kidney, OCT2, PRRX1, and SOX2 expression is significantly deregulated in primary ccRCC. When ccRCCs are stratified by their SETD2 mutation status, we observe that expression of OCT2, PRRX1, and SOX2 is higher in SETD2 mutant compared to WT ccRCC (Fig. S8C), consistent with findings in the RPTEC model. Furthermore, higher expression of all three genes is associated with ccRCC metastasis and significantly worse overall survival (Fig. S8D,E). Collectively these data demonstrate SOX2, PRRX1, and OCT2 are downstream effectors of SETD2 EMT and stemness pathways that are also relevant to ccRCC patient outcome. Thus, future therapeutics directed against these genes, or their pathways, may represent individualized therapeutics that would more efficiently target SETD2‐mutant ccRCC.

## 4 Discussion

Previous studies have shown that SETD2 mutations correlate with high stage and grade, and higher risk of recurrence in ccRCC patients, indicating that SETD2 and H3K36me3 play a role in driving invasion and early metastasis [2, 46]. The mechanisms underlying these observations, however, have remained elusive. Using nontumorigenic RPTEC that lack VHL, PBRM1, and BAP1 mutations as a model reveals that SETD2 loss drives pro‐tumorigenic expression signatures including TGF‐β‐independent EMT and other unique pathways including inflammation, secreted factors, and stemness. Our data demonstrate that SETD2 and its downstream targets (SOX2, OCT2, and PRRX1) are key regulators of EMT and stemness, consistent with SETD2 loss promoting more aggressive ccRCC, and that these phenotypic effects are driven by both cell intrinsic and extrinsic mechanisms (Fig. 7E). Furthermore, the EMT‐related phenotypes are largely reversible with SETD2 rescue. Our findings are overall consistent with a recent study from Xie et al. [5], where the SETD2‐deficient JHRCC12 ccRCC cell line was rescued with an N‐terminally truncated SETD2 isoform, which restored H3K36me3 levels and suppressed metastases in a xenograft model. Our observation that SETD2 loss drives an inflammatory gene expression signature accompanied by cytokine secretion is also consistent with a recent study of MLL3, a histone methyltransferase mutated in many cancers. In this study, MLL3 loss promoted EMT and stemness in part through deregulated interferon signaling [38].

The dramatic alteration in expression patterns that include both up‐ and downregulated genes, despite the overall profound loss in H3K36me3, could be driven by changes in H3K36me3 itself or through changes in levels or redistribution of other histone marks. Crosstalk between methylation at the H3K36 and H3K27 positions, for example, could in part explain the widespread changes in expression [10]. In general, methylation at these positions is thought to be antagonistic via their interplay at the level of K27/K36 dimethylation [47, 48]. DNA methylation, which is recruited by H3K36me2/me3, may redistribute upon SETD2 KO and influence enhancer/promoter activity [23]. Broader profiling of the epigenome of SETD2‐deficient cells will clarify the mechanisms involved and provide clues as to why restoration of SETD2 only partially rescues the transcriptome.

Observations from ITH studies in ccRCC revealed that SETD2 mutations are independently acquired in multiple regions of individual tumors [8], indicating that loss of H3K36me3 actively confers some advantage onto tumor cells. The competitive and/or cooperative interactions between SETD2‐deficient cells and other tumor subclones that result from ITH, and how these interactions impact growth properties of neighboring cancer cells or the tumor as a whole through non‐cell autonomous mechanisms remains to be determined. Examples of such paracrine interactions include glioblastoma, where cancer cells expressing mutant EGFR stimulate the growth of tumor cells with WT EGFR through cytokine secretion (IL‐6 and LIF) [49]. Moreover, in medulloblastoma, heterogeneous inactivation of the PRC2 complex results in a subpopulation of cells that are less fit, but secrete IGF2 that enhances overall tumor growth [50]. Given these examples, the paracrine signaling effects we observed where CM from SETD2 KO RPTEC stimulates migration and invasion of SETD2 WT cells may explain how SETD2 deficiency contributes to heterogeneity and promotes metastasis (despite SETD2‐deficient cells proliferating less rapidly). A minor proportion of cancer cells with a mutation in SETD2 may secrete factors into the TME that favor growth or invasion of the tumor collectively. A key future direction will include identifying the active paracrine factor(s) and examining whether this mechanism can be therapeutically targeted to reduce metastatic potential of SETD2‐mutant ccRCC.

There are several limitations to our study. While on the one hand the lack of other SETD2 co‐occurring mutations in ccRCC such as VHL or PBRM1 in our model allows for more precise definition of functions attributable directly to SETD2, it does not consider how SETD2 activities might be modified by the presence of other mutations. Building such combinatorial models is feasible with CRISPR‐based methods. Another limitation is that we do not establish whether SETD2 loss is tumorigenic or modifies *in vivo* tumor metastatic properties. Such studies, which can be addressed through xenograft models, would likely also require the introduction of one or more of the common SETD2 co‐occurring mutations in ccRCC such as VHL and/or PBRM1, as noted above. Whether or not SETD2 loss alone transforms RPTEC does not diminish the importance of SETD2 regulation of EMT and its role in early metastasis. Indeed, although combined deletion of Vhl and Pbrm1 results in multifocal clear cell kidney cancers indicating that Setd2 inactivation is not required for kidney tumor initiation, tumors did not metastasize in this model [51] and loss of SETD2 may be an essential step in this process. In addition, SETD2 is mutated in other cancer types, such as non‐small cell lung cancer, where VHL and PBRM1 mutations rarely occur, although this does not preclude the possibility that SETD2 cooperates with other mutated genes such as K‐RAS in context of lung cancer.

## 5 Conclusions

Using SETD2 isogenic RPTEC lines as a model, we showed that the EMT pathway is significantly upregulated upon SETD2 inactivation or with TGF‐β treatment of WT cells. The molecular profile of the SETD2‐driven EMT program is distinct from the normal EMT program induced by TGF‐β. Many cytokines and chemokines are regulated by SETD2, suggesting the EMT is partially mediated by paracrine signaling. Phenotypically, SETD2 KO cells are more migratory and invasive than WT RPTEC, indicating loss of SETD2 drives invasion and likely metastasis. The increased invasiveness is consistent with patient data of SETD2 mutations correlating with poor survival and increased metastatic recurrence. Furthermore, SETD2 WT cells treated with SETD2 KO CM are more aggressive than when exposed to parental RPTEC‐derived CM, demonstrating that secreted factors in CM are another factor contributing to the SETD2‐driven EMT program. Ectopic re‐expression of SETD2 in SETD2 KO allows the partial rescue of transcriptome changes and complete reversal of global H3K36me3 loss. By analyzing ATAC‐seq data, we identified SOX2, PRRX1, and OCT2 as key TFs involved in the SETD2 loss‐driven EMT program. These three genes are uniquely induced in the RPTEC SETD2 KO cells, and their expression is reversed in rescue cells. Ectopic expression of SOX2, PRRX1, or OCT2 in WT RPTEC largely recapitulates the EMT phenotypes of increased migration, invasion, and stemness observed in SETD2 KO RPTEC, indicating that they contribute to the SETD2 loss‐driven EMT program. Furthermore, high expression of SOX2, PRRX1, or OCT2 correlates with poor patient outcome and worse survival rate, and therefore these three genes could be therapeutic targets for SETD2‐mutant ccRCC patients.

## Conflict of interest

THH: Advisory board participation: Surface Therapeutics, Exelixis, Genentech, Pfizer, Ipsen, Cardinal Health; research support‐Novartis. The remaining authors declare no conflict of interest.

## Author contributions

KDR and THH contributed to study design. TW performed most experiments and analyzed the data. RTW contributed to cloning, validation, and lentiviral transduction for various cell line models used in the paper. SK performed TCGA analysis. HL, EPC, and DFL contributed to derivation of a cell line model. XP and XZ performed a subset of experiments. J‐HL contributed to ChIP‐seq and ATAC‐seq. TW, RAH, and LW contributed to bioinformatics analysis of RNA‐seq, ChIP‐seq, and ATAC‐seq data. KDR and TW prepared the manuscript. KDR, THH, RTW, RAH, and TW reviewed the manuscript.

## Supporting information


**Fig. S1.** Summary of methods to study SETD2 loss‐driven EMT program.
**Fig. S2.** SETD2 loss drives changes in epithelial, mesenchymal, and stemness markers by immunofluorescence microscopy.
**Fig. S3.** SETD2 loss‐of‐function uniquely modulates key growth pathways that are consistent with analysis of TCGA ccRCC tumor samples.
**Fig. S4.** Impact of SETD2 status on cell growth rate and stemness gene expression patterns.
**Fig. S5.** SETD2 KO enhances migration in ccRCC cell line models.
**Fig. S6.** Chromatin is overall less accessible at epithelial genes but more open at mesenchymal genes in SETD2 KO cells.
**Fig. S7.** Transcription factor footprinting highlights SETD2 deregulated transcription factors.
**Fig. S8.** Expression of SETD2 effector genes SOX2, OCT2, and PRRX1 identified in the RPTEC model correlates with poor outcome in TCGA‐KIRC ccRCC patients.

**Table S1.** Primer sequences.

**Table S2.** Summary of sequencing quality.

**Table S3.** Subset of TCGA KIRC samples used in this study.

**Table S4.** RPTEC TGF‐β and SETD2 KO differentially expressed genes.

**Table S5.** Summary of growth rate data for isogenic cell line models.

**Table S6.** Expression data for RPTEC WT, SETD2 KO, and rescue for the NABA‐secreted factors pathway.

**Table S7.** MEME motif search results from the differential ATAC‐seq analysis.

**Table S8.** RPTEC SETD2‐mutant vs WT differential binding score and p‐value output from TOBIAS analysis of ATAC‐seq data.

## Data Availability

RNA‐seq, ChIP‐seq, and ATAC‐seq data generated in this study are available for download in NCBI GEO accession GSE213260.

## References

[1] Young AP , Schlisio S , Minamishima YA , Zhang Q , Li L , Grisanzio C , et al. VHL loss actuates a HIF‐independent senescence programme mediated by Rb and p400. Nat Cell Biol. 2008;10(3):361–369. 18297059 10.1038/ncb1699

[2] Ho TH , Choueiri TK , Wang K , Karam JA , Chalmers Z , Frampton G , et al. Correlation between molecular subclassifications of clear cell renal cell carcinoma and targeted therapy response. Eur Urol Focus. 2016;2(2):204–209. 28723536 10.1016/j.euf.2015.11.007

[3] Simon JM , Hacker KE , Singh D , Brannon AR , Parker JS , Weiser M , et al. Variation in chromatin accessibility in human kidney cancer links H3K36 methyltransferase loss with widespread RNA processing defects. Genome Res. 2014;24(2):241–250. 24158655 10.1101/gr.158253.113 PMC3912414

[4] Ricketts CJ , de Cubas AA , Fan H , Smith CC , Lang M , Reznik E , et al. The Cancer Genome Atlas comprehensive molecular characterization of renal cell carcinoma. Cell Rep. 2018;23(1):313–326.e5. 29617669 10.1016/j.celrep.2018.03.075 PMC6075733

[5] Xie Y , Sahin M , Sinha S , Wang Y , Nargund AM , Lyu Y , et al. SETD2 loss perturbs the kidney cancer epigenetic landscape to promote metastasis and engenders actionable dependencies on histone chaperone complexes. Nat Cancer. 2022;3:188–202. 35115713 10.1038/s43018-021-00316-3 PMC8885846

[6] van der Mijn JC , Eng KW , Chandra P , Fernandez E , Ramazanoglu S , Sigaras A , et al. The genomic landscape of metastatic clear cell renal cell carcinoma after systemic therapy. Mol Oncol. 2022;16:2384–2395. 35231161 10.1002/1878-0261.13204 PMC9208073

[7] Bihr S , Ohashi R , Moore AL , Rüschoff JH , Beisel C , Hermanns T , et al. Expression and mutation patterns of PBRM1, BAP1 and SETD2 mirror specific evolutionary subtypes in clear cell renal cell carcinoma. Neoplasia. 2019;21(2):247–256. 30660076 10.1016/j.neo.2018.12.006 PMC6355619

[8] Gerlinger M , Rowan AJ , Horswell S , Math M , Larkin J , Endesfelder D , et al. Intratumor heterogeneity and branched evolution revealed by multiregion sequencing. N Engl J Med. 2012;366(10):883–892. 22397650 10.1056/NEJMoa1113205 PMC4878653

[9] Niu N , Lu P , Yang Y , He R , Zhang L , Shi J , et al. Loss of Setd2 promotes Kras‐induced acinar‐to‐ductal metaplasia and epithelia‐mesenchymal transition during pancreatic carcinogenesis. Gut. 2019;69:715–726. 31300513 10.1136/gutjnl-2019-318362

[10] Yuan H , Han Y , Wang X , Li N , Liu Q , Yin Y , et al. SETD2 restricts prostate cancer metastasis by integrating EZH2 and AMPK signaling pathways. Cancer Cell. 2020;38:350–365.e7. 32619406 10.1016/j.ccell.2020.05.022

[11] Yang X , Chen R , Chen Y , Zhou Y , Wu C , Li Q , et al. Methyltransferase SETD2 inhibits tumor growth and metastasis via STAT1–IL‐8 signaling‐mediated epithelial–mesenchymal transition in lung adenocarcinoma. Cancer Sci. 2022;113:1195–1207. 35152527 10.1111/cas.15299 PMC8990294

[12] Mar BG , Bullinger LB , McLean KM , Grauman PV , Harris MH , Stevenson K , et al. Mutations in epigenetic regulators including SETD2 are gained during relapse in paediatric acute lymphoblastic leukaemia. Nat Commun. 2014;5:3469. 24662245 10.1038/ncomms4469 PMC4016990

[13] Simeonov KP , Byrns CN , Clark ML , Norgard RJ , Martin B , Stanger BZ , et al. Single‐cell lineage tracing of metastatic cancer reveals selection of hybrid EMT states. Cancer Cell. 2021;39(8):1150–1162.e9. 34115987 10.1016/j.ccell.2021.05.005 PMC8782207

[14] Miettinen PJ , Ebner R , Lopez AR , Derynck R . TGF‐beta induced transdifferentiation of mammary epithelial cells to mesenchymal cells: involvement of type I receptors. J Cell Biol. 1994;127(6 Pt 2):2021–2036. 7806579 10.1083/jcb.127.6.2021 PMC2120317

[15] Piek E , Moustakas A , Kurisaki A , Heldin CH , Dijke P . TGF‐(beta) type I receptor/ALK‐5 and Smad proteins mediate epithelial to mesenchymal transdifferentiation in NMuMG breast epithelial cells. J Cell Sci. 1999;112(Pt 24):4557–4568. 10574705 10.1242/jcs.112.24.4557

[16] Valcourt U , Kowanetz M , Niimi H , Heldin CH , Moustakas A . TGF‐beta and the Smad signaling pathway support transcriptomic reprogramming during epithelial‐mesenchymal cell transition. Mol Biol Cell. 2005;16(4):1987–2002. 15689496 10.1091/mbc.E04-08-0658 PMC1073677

[17] Xu J , Lamouille S , Derynck R . TGF‐beta‐induced epithelial to mesenchymal transition. Cell Res. 2009;19(2):156–172. 19153598 10.1038/cr.2009.5 PMC4720263

[18] Pastushenko I , Brisebarre A , Sifrim A , Fioramonti M , Revenco T , Boumahdi S , et al. Identification of the tumour transition states occurring during EMT. Nature. 2018;556(7702):463–468. 29670281 10.1038/s41586-018-0040-3

[19] Williams ED , Gao D , Redfern A , Thompson EW . Controversies around epithelial–mesenchymal plasticity in cancer metastasis. Nat Rev Cancer. 2019;19(12):716–732. 31666716 10.1038/s41568-019-0213-x PMC7055151

[20] Lambert AW , Weinberg RA . Linking EMT programmes to normal and neoplastic epithelial stem cells. Nat Rev Cancer. 2021;21(5):325–338. 33547455 10.1038/s41568-021-00332-6

[21] Kolasinska‐Zwierz P , Down T , Latorre I , Liu T , Liu XS , Ahringer J . Differential chromatin marking of introns and expressed exons by H3K36me3. Nat Genet. 2009;41(3):376–381. 19182803 10.1038/ng.322 PMC2648722

[22] Tiedemann RL , Hlady RA , Hanavan PD , Lake DF , Tibes R , Lee JH , et al. Dynamic reprogramming of DNA methylation in SETD2‐deregulated renal cell carcinoma. Oncotarget. 2015;7(2):1927–1946. 10.18632/oncotarget.6481 PMC4811507 26646321

[23] Weinberg DN , Papillon‐Cavanagh S , Chen H , Yue Y , Chen X , Rajagopalan KN , et al. The histone mark H3K36me2 recruits DNMT3A and shapes the intergenic DNA methylation landscape. Nature. 2019;573:281–286. 31485078 10.1038/s41586-019-1534-3 PMC6742567

[24] Pfister SX , Ahrabi S , Zalmas LP , Sarkar S , Aymard F , Bachrati CZ , et al. SETD2‐dependent histone H3K36 trimethylation is required for homologous recombination repair and genome stability. Cell Rep. 2014;7(6):2006–2018. 24931610 10.1016/j.celrep.2014.05.026 PMC4074340

[25] Carvalho S , Raposo AC , Martins FB , Grosso AR , Sridhara SC , Rino J , et al. Histone methyltransferase SETD2 coordinates FACT recruitment with nucleosome dynamics during transcription. Nucleic Acids Res. 2013;41(5):2881–2893. 23325844 10.1093/nar/gks1472 PMC3597667

[26] Fifield AL , Hanavan PD , Faigel DO , Sergienko E , Bobkov A , Meurice N , et al. Molecular inhibitor of QSOX1 suppresses tumor growth in vivo. Mol Cancer Ther. 2020;19(1):112–122. 31575656 10.1158/1535-7163.MCT-19-0233 PMC6946859

[27] Thompson JJ , Kaur R , Sosa CP , Lee JH , Kashiwagi K , Zhou D , et al. ZBTB24 is a transcriptional regulator that coordinates with DNMT3B to control DNA methylation. Nucleic Acids Res. 2018;46(19):10034–10051. 30085123 10.1093/nar/gky682 PMC6212772

[28] Justus CR , Leffler N , Ruiz‐Echevarria M , Yang LV . In vitro cell migration and invasion assays. J Vis Exp. 2014;88:51046. 10.3791/51046 PMC4186330 24962652

[29] Hlady RA , Zhao X , el Khoury LY , Luna A , Pham K , Wu Q , et al. Interferon drives HCV scarring of the epigenome and creates targetable vulnerabilities following viral clearance. Hepatology. 2022;75(4):983–996. 34387871 10.1002/hep.32111 PMC9416882

[30] Lenkiewicz E , Malasi S , Hogenson TL , Flores LF , Barham W , Phillips WJ , et al. Genomic and epigenomic landscaping defines new therapeutic targets for adenosquamous carcinoma of the pancreas. Cancer Res. 2020;80(20):4324–4334. 32928922 10.1158/0008-5472.CAN-20-0078 PMC7906529

[31] Patro R , Duggal G , Love MI , Irizarry RA , Kingsford C . Salmon provides fast and bias‐aware quantification of transcript expression. Nat Methods. 2017;14(4):417–419. 28263959 10.1038/nmeth.4197 PMC5600148

[32] Bentsen M , Goymann P , Schultheis H , Klee K , Petrova A , Wiegandt R , et al. ATAC‐seq footprinting unravels kinetics of transcription factor binding during zygotic genome activation. Nat Commun. 2020;11(1):4267. 32848148 10.1038/s41467-020-18035-1 PMC7449963

[33] Wieser M , Stadler G , Jennings P , Streubel B , Pfaller W , Ambros P , et al. hTERT alone immortalizes epithelial cells of renal proximal tubules without changing their functional characteristics. Am J Physiol Renal Physiol. 2008;295(5):F1365–F1375. 18715936 10.1152/ajprenal.90405.2008

[34] Zhang Y , Narayanan SP , Mannan R , Raskind G , Wang X , Vats P , et al. Single‐cell analyses of renal cell cancers reveal insights into tumor microenvironment, cell of origin, and therapy response. Proc Natl Acad Sci USA. 2021;118(24):e2103240118. 34099557 10.1073/pnas.2103240118 PMC8214680

[35] Yoshikawa M , Hishikawa K , Marumo T , Fujita T . Inhibition of histone deacetylase activity suppresses epithelial‐to‐mesenchymal transition induced by TGF‐β1 in human renal epithelial cells. J Am Soc Nephrol. 2007;18(1):58–65. 17135397 10.1681/ASN.2005111187

[36] Dudas PL , Argentieri RL , Farrell FX . BMP‐7 fails to attenuate TGF‐beta1‐induced epithelial‐to‐mesenchymal transition in human proximal tubule epithelial cells. Nephrol Dial Transplant. 2009;24(5):1406–1416. 19056781 10.1093/ndt/gfn662

[37] Li H‐T , Jang HJ , Rohena‐Rivera K , Liu M , Gujar H , Kulchycki J , et al. RNA mis‐splicing drives viral mimicry response after DNMTi therapy in SETD2‐mutant kidney cancer. Cell Rep. 2023;42(1):112016. 36662621 10.1016/j.celrep.2023.112016 PMC10034851

[38] Mani SA , Guo W , Liao MJ , Eaton EN , Ayyanan A , Zhou AY , et al. The epithelial‐mesenchymal transition generates cells with properties of stem cells. Cell. 2008;133(4):704–715. 18485877 10.1016/j.cell.2008.03.027 PMC2728032

[39] Fiedorowicz M , Khan MI , Strzemecki D , Orzeł J , Wełniak‐Kamińska M , Sobiborowicz A , et al. Renal carcinoma CD105−/CD44− cells display stem‐like properties in vitro and form aggressive tumors in vivo. Sci Rep. 2020;10(1):5379. 32214151 10.1038/s41598-020-62205-6 PMC7096525

[40] Zhu Y , Huang S , Chen S , Chen J , Wang Z , Wang Y , et al. SOX2 promotes chemoresistance, cancer stem cells properties, and epithelial‐mesenchymal transition by beta‐catenin and Beclin1/autophagy signaling in colorectal cancer. Cell Death Dis. 2021;12(5):449. 33953166 10.1038/s41419-021-03733-5 PMC8100126

[41] Du W , Liu X , Yang M , Wang W , Sun J . The regulatory role of PRRX1 in cancer epithelial‐mesenchymal transition. Onco Targets Ther. 2021;14:4223–4229. 34295164 10.2147/OTT.S316102 PMC8291965

[42] Wang S‐M , Tie J , Wang WL , Hu SJ , Yin JP , Yi XF , et al. POU2F2‐oriented network promotes human gastric cancer metastasis. Gut. 2016;65(9):1427–1438. 26019213 10.1136/gutjnl-2014-308932 PMC5036257

[43] Walter KR , Balko JM , Hagan CR . Progesterone receptor promotes degradation of STAT2 to inhibit the interferon response in breast cancer. Oncoimmunology. 2020;9(1):1758547. 32391191 10.1080/2162402X.2020.1758547 PMC7199813

[44] Marta ZN , Agnieszka W , Jacek P , Jeleń A , Adrian K , Dagmara SK , et al. NFKB2 gene expression in patients with peptic ulcer diseases and gastric cancer. Mol Biol Rep. 2020;47(3):2015–2021. 32056043 10.1007/s11033-020-05299-5

[45] Ji Z , He L , Regev A , Struhl K . Inflammatory regulatory network mediated by the joint action of NF‐kB, STAT3, and AP‐1 factors is involved in many human cancers. Proc Natl Acad Sci USA. 2019;116(19):9453–9462. 30910960 10.1073/pnas.1821068116 PMC6511065

[46] Ho TH , Park IY , Zhao H , Tong P , Champion MD , Yan H , et al. High‐resolution profiling of histone h3 lysine 36 trimethylation in metastatic renal cell carcinoma. Oncogene. 2016;35(12):1565–1574. 26073078 10.1038/onc.2015.221 PMC4679725

[47] Schmitges FW , Prusty AB , Faty M , Stützer A , Lingaraju GM , Aiwazian J , et al. Histone methylation by PRC2 is inhibited by active chromatin marks. Mol Cell. 2011;42(3):330–341. 21549310 10.1016/j.molcel.2011.03.025

[48] Streubel G , Watson A , Jammula SG , Scelfo A , Fitzpatrick DJ , Oliviero G , et al. The H3K36me2 methyltransferase Nsd1 demarcates PRC2‐mediated H3K27me2 and H3K27me3 domains in embryonic stem cells. Mol Cell. 2018;70(2):371–379.e5. 29606589 10.1016/j.molcel.2018.02.027

[49] Inda M‐M , Bonavia R , Mukasa A , Narita Y , Sah DWY , Vandenberg S , et al. Tumor heterogeneity is an active process maintained by a mutant EGFR‐induced cytokine circuit in glioblastoma. Genes Dev. 2010;24(16):1731–1745. 20713517 10.1101/gad.1890510 PMC2922502

[50] Yi J , Kim BW , Shi X , Zhan X , Lu QR , Xuan Z , et al. PRC2 heterogeneity drives tumor growth in medulloblastoma. Cancer Res. 2022;82(16):2874–2886. 35731926 10.1158/0008-5472.CAN-21-4313 PMC9388591

[51] Nargund AM , Pham CG , Dong Y , Wang PI , Osmangeyoglu HU , Xie Y , et al. The SWI/SNF protein PBRM1 restrains VHL‐loss‐driven clear cell renal cell carcinoma. Cell Rep. 2017;18(12):2893–2906. 28329682 10.1016/j.celrep.2017.02.074 PMC5431084

