# Supplementary material for: SETD2 loss in renal epithelial cells drives epithelial‐to‐mesenchymal transition in a TGF‐β‐independent manner
Source: Mol Oncol. 2023 Jul 17;18(1):44–61. doi: 10.1002/1878-0261.13487 (PMC10766198; doi:10.1002/1878-0261.13487)
Supplement: Supplementary file 1 — Fig. S1. Summary of methods to study SETD2 loss‐driven EMT program. Fig. S2. SETD2 loss drives changes in epithelial, mesenchymal, and stemness markers by immunofluorescence microscopy. Fig. S3. SETD2 loss‐of‐function uniquely modulates key growth pathways that are consistent with analysis of TCGA ccRCC tumor samples. Fig. S4. Impact of SETD2 status on cell growth rate and stemness gene expression patterns. Fig. S5. SETD2 KO enhances migration in ccRCC cell line models. Fig. S6. Chromatin is overall less accessible at epithelial genes but more open at mesenchymal genes in SETD2 KO cells. Fig. S7. Transcription factor footprinting highlights SETD2 deregulated transcription factors. Fig. S8. Expression of SETD2 effector genes SOX2, OCT2, and PRRX1 identified in the RPTEC model correlates with poor outcome in TCGA‐KIRC ccRCC patients. [file MOL2-18-44-s006.pdf]

Supplemental material for Wang *et al.*, SETD2 loss in renal epithelial cells drives epithelial-to-mesenchymal transition in a TGF-β independent manner

Supplemental Data

Supplemental Table S1: Primer sequences.

Supplemental Table S2: Summary of sequencing quality.

Supplemental Table S3: Subset of TCGA KIRC samples used in this study.

Supplemental Table S4: RPTEC TGFβ and SETD2 KO differentially expressed genes.

Supplemental Table S5: Summary of growth rate data for isogenic cell line models.

Supplemental Table S6: Expression data for RPTEC WT, SETD2 KO, and rescue for the NABA secreted factors pathway.

Supplemental Table S7: MEME motif search results from the differential ATAC-seq analysis.

Supplemental Table S8: RPTEC SETD2 mutant vs WT differential binding score and p-value output from TOBIAS analysis of ATAC-seq data.

Supplementary Figures: S1-S8

Supplemental Figure Legends

Figure S1: Summary of methods to study SETD2 loss driven EMT program. (A) Overall approach to characterize SETD2 loss-driven EMT program. **(B)** Schematic of the SETD2 genomic locus on 3p21 with a blow-up of the exon 3 region targeted by the guide RNA and sequencing primers used to confirm SETD2 mutations. Sanger sequencing results of two independent KO clones. KO1 has 1 bp and 5 bp deletions, which results in novel amino acid sequence (indicated by red) and a stop codon (indicated by white asterisks in red squares). For the -1 allele, there are novel 60 amino acid sequences after frameshift before a new stop codon. KO2 has a 14bp deletion and 1bp insertion leading to a frameshift mutation. Stop codons are indicated by white asterisks in red squares.

Figure S2: SETD2 loss drives changes in epithelial, mesenchymal, and stemness markers by immunofluorescence microscopy. Immunofluorescence staining of (A) epithelial (CDH1, MUC1), (B) mesenchymal (MMP2, SNAI2), and (C) stemness (CD44) markers in the RPTEC parent and SETD2 KO1 and KO2 clones (green channel). Representative images are shown with DAPI DNA counterstain (dlue channel) demonstrating a shift towards stemness and mesenchymal expression, and away from epithelial gene expression in SETD2 KO RPTEC. Scale bar is shown, images are taken at 40X magnification.

Figure S3: **SETD2 loss-of-function uniquely modulates key growth pathways that are consistent with analysis of TCGA ccRCC tumor samples. (A)** Principal component analysis of all genes representing global expression patterns in 12 SETD2 WT samples and 12 SETD2 mut samples derived from TCGA's KIRC dataset (as described in Methods). **(B)** Volcano plot of differentially expressed genes between SETD2 mutant and SETD2 WT ccRCCs tissue samples in panel B. **(C)** Heatmap of the differentially expressed genes derived from TCGA samples in

(B) plotted for RPTEC WT (blue), SETD2 KO (red), and 3XFLAG-SETD2 KO rescue cells (green), each in duplicate. **(D)** A scatterplot of differentially expressed genes from (B) plotted using RPTEC KO vs WT and RPTEC rescue vs KO comparisons, demonstrating both confirmation of the SETD2 expression signature in RPTEC cells, and concomittant reversal of the phenotype with ectopic re-expression.

Figure S4: Impact of SETD2 status on cell growth rate and stemness gene expression patterns. (A) Cell growth curve for the indicated RPTEC lines under 10% serum standard growth conditions (left) and 2% low serum conditions used for wound healing migration assays (right). Doubling times in hours are indicated in each graph and are also summarized in Supplemental Table S5. **(B)** RT-qPCR results comparing stemness gene expression between RPTEC WT and SETD2 KO clones. One-way ANOVA is used for statistical test. ****, $p<0.0001$; ***, $p<0.001$; **, $p<0.01$; *, $p<0.05$.

Figure S5: SETD2 KO enhances migration in ccRCC cell line models. (A) Sanger sequencing confirmation of the frameshift mutations in SETD2 for the RCJ-41T1 (top right panel) cell line model (top). **(B)** Western blotting for H3K36me1/2/3 levels in 786-O (Tiedemann et al., 2016) and RCJ-41T1 (Fifield et al., 2020) SETD2 WT and SETD2 KO cell line models (bottom). **(C)** Wound healing assay to test migration of SETD2 KO cells in 786-O and RCJ-T1 models. Quantification of results are shown in the graph at the right. P-value is calculated using t-test. Magnification, 4x. Scale bar, 1000μm. **, $p<0.01$. **(D)** Transwell assay testing invasiveness of the indicated ccRCC cell lines. Representative images of crystal violet-stained cells that invaded through the membrane are shown beside the graphs. ****, $p<0.0001$.

Figure S6: Chromatin is overall less accessible at epithelial genes but more open at mesenchymal genes in SETD2 KO cells. (A) Representative genome browser views for

ATAC-seq signals in EMT-related genes in the three isogenic RPTEC lines. Epithelial genes are shown at the top. A mesenchymal gene is shown on the bottom. GeneHancer derived promoters and enhancers are indicated by red and gray lines, respectively. **(B)** Tag density plots showing overall chromatin accessibility differences at epithelial genes (top panel) and mesenchymal genes (bottom panel) in RPTEC WT, SETD2 KO, and SETD2 KO rescue cells. Epithelial and mesenchymal gene sets are derived from [6], as described in the main text. ATAC-seq signal is displayed as reads per genome coverage (RPGC).

Figure S7: Transcription factor footprinting highlights SETD2 deregulated transcription factors. (A) A volcano plot of differential binding scores against significance from the TOBIAS [5] output highlighting the top 10% decreased (green) and increased (red) differential binding scores in SETD2 KO vs WT RPTEC cells from ATAC-seq data. **(B)** A heatmap of the 532 target genes of SOX2, POU2F2, and PRRX1 with expression data in RPTEC KO and WT cells. 'A/B' refer to replicates. **(C)** A network plot of the twenty most differentially upregulated genes linked to each of the three transcription factors (SOX2, PRRX1, and POU2F2 hubs, colored in blue). A color scale is shown.

Figure S8: Expression of SETD2 effector genes SOX2, OCT2 and PRRX1 identified in the RPTEC model correlates with poor outcome in TCGA-KIRC ccRCC patients. (A) Western blot showing endogenous expression level of SOX2, OCT2, and PRRX1 in SETD2 WT, KO, rescue and TGFβ-treated WT RPTEC cells. **(B)** Western blot validating ectopic expression of the three candidate SETD2 effector genes in WT RPTEC cells. **(C)** Expression of the three genes in TCGA-KIRC normal kidney and ccRCC samples, where the tumor samples are stratified by SETD2 mutational status. **(D)** Expression of the indicated genes in TCGA-KIRC ccRCC non-metastatic samples (M0) compared to metastatic samples (M1). **(E)** Overall survival rate of of ccRCC patients according to SOX2, OCT2, and PRRX1 expression using TCGA

ccRCC tumor samples. P-value is calculated using Wilcoxon signed-rank test in C) and D) and log-rank test in E).

Supplemental References

1. Fifield, A.L., et al., Molecular inhibitor of QSOX1 suppresses tumor growth in vivo*. Mol Cancer Ther*, 2020. **19**(1): p. 112-122.
2. Thompson, J.J., et al., ZBTB24 is a transcriptional regulator that coordinates with DNMT3B to control DNA methylation*. Nucleic Acids Res*, 2018. **46**(19): p. 10034-10051.
3. Hlady, R.A., et al., Interferon drives HCV scarring of the epigenome and creates targetable vulnerabilities following viral clearance*. Hepatology*, 2022. **75**(4):983-996.
4. Patro, R., et al., Salmon provides fast and bias-aware quantification of transcript expression*. Nat Methods*, 2017. **14**(4): p. 417-419.
5. Bentsen, M., et al., ATAC-seq footprinting unravels kinetics of transcription factor binding during zygotic genome activation*. Nature Comm*, 2020. **11**(1): p. 4267.
6. Simeonov, K.P., et al., Single-cell lineage tracing of metastatic cancer reveals selection of hybrid EMT states*. Cancer Cell*, 2021. **39**(8): p. 1150-1162.e9.

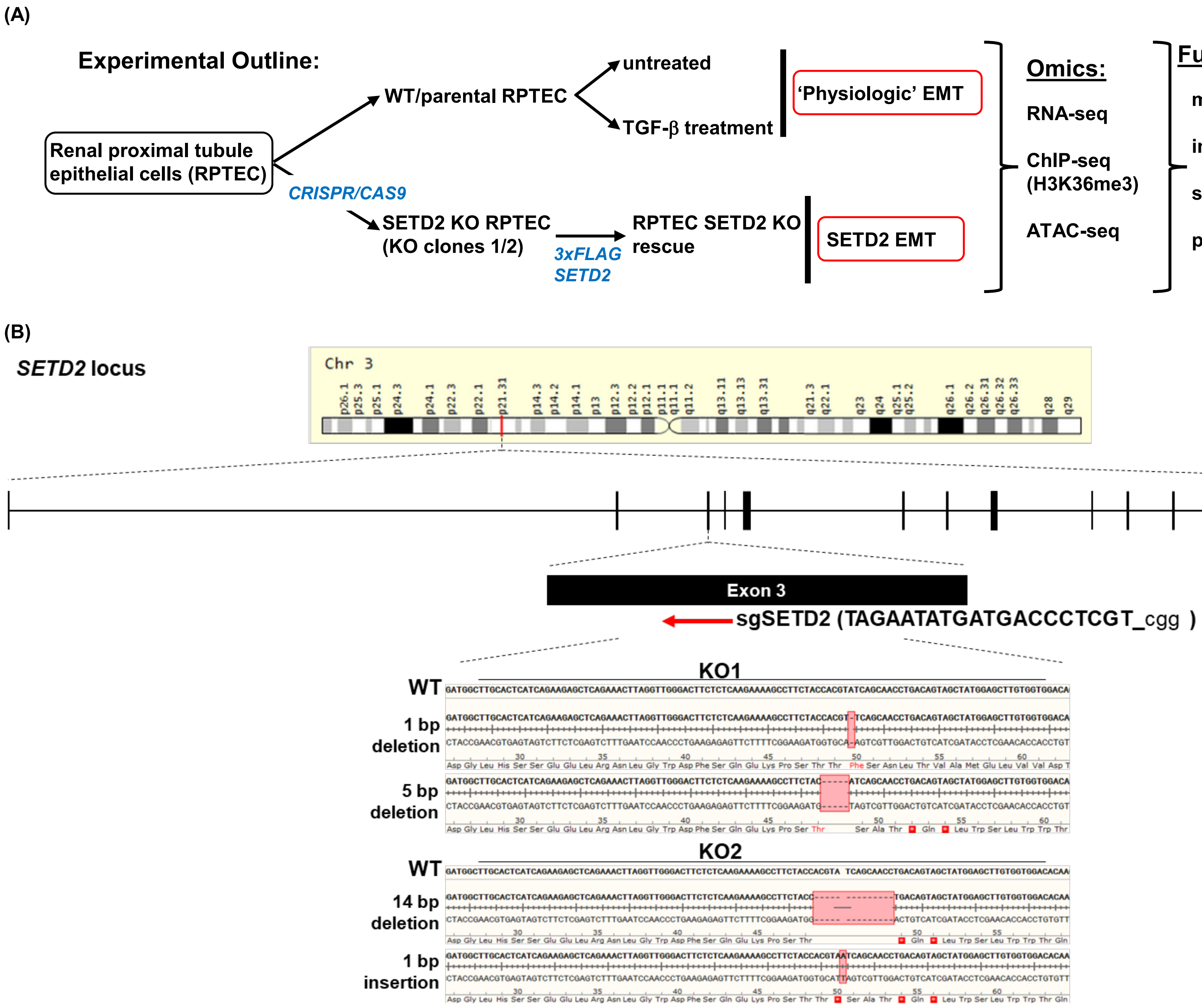

Supplemental Figure S1

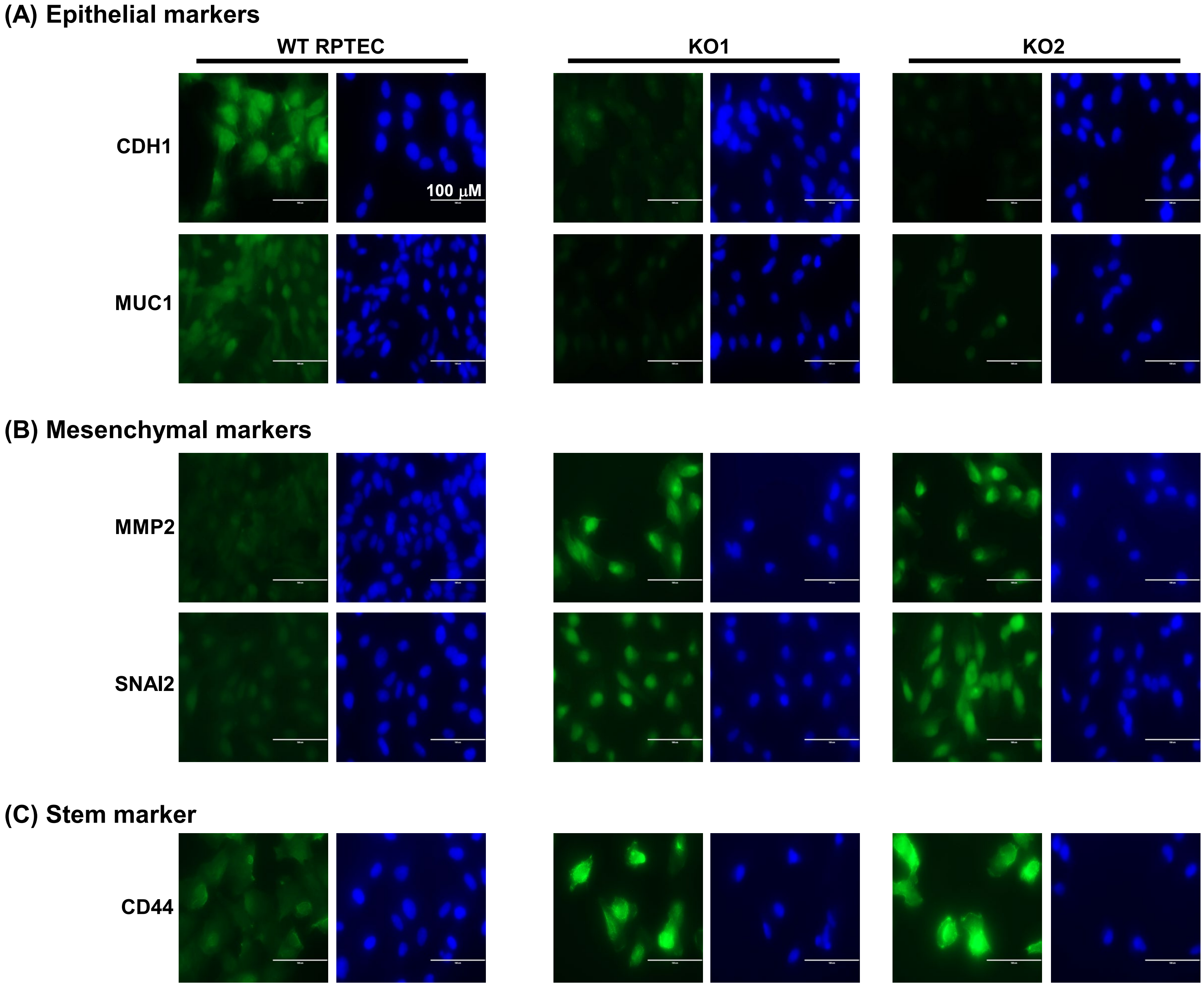

Supplemental Figure S2

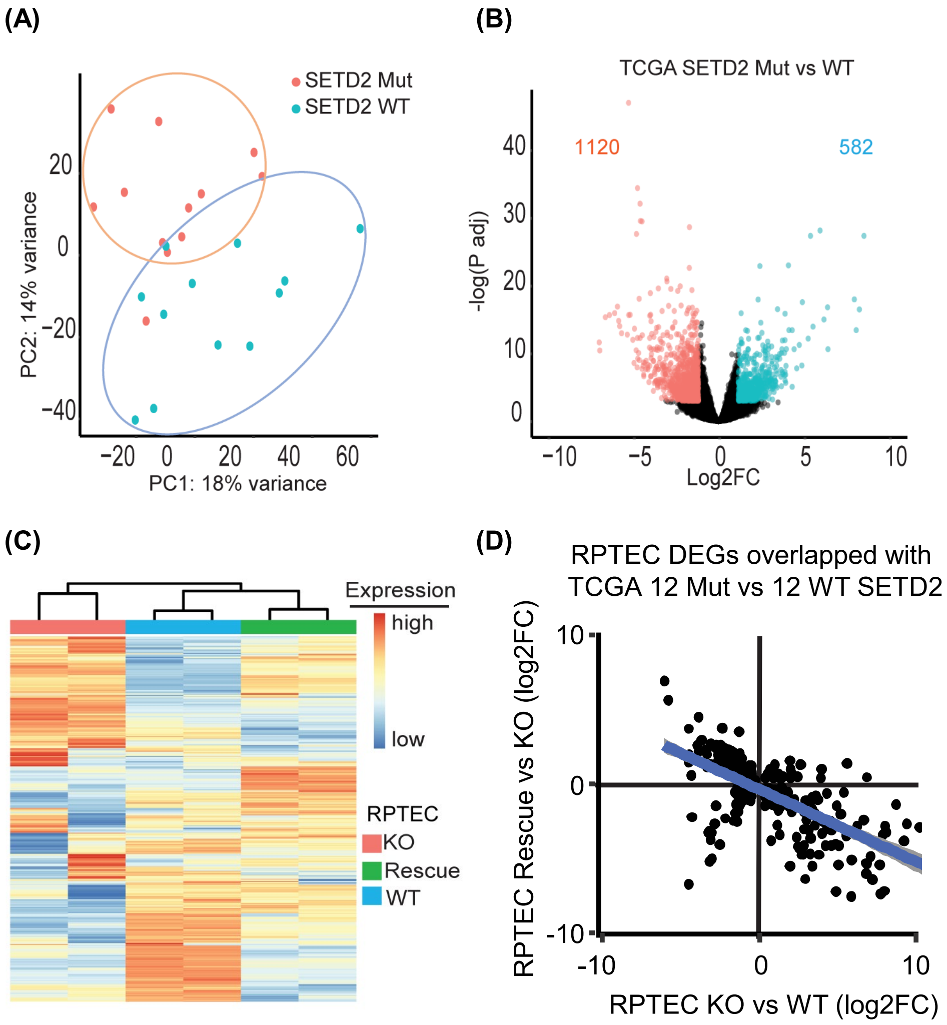

Supplemental Figure S3

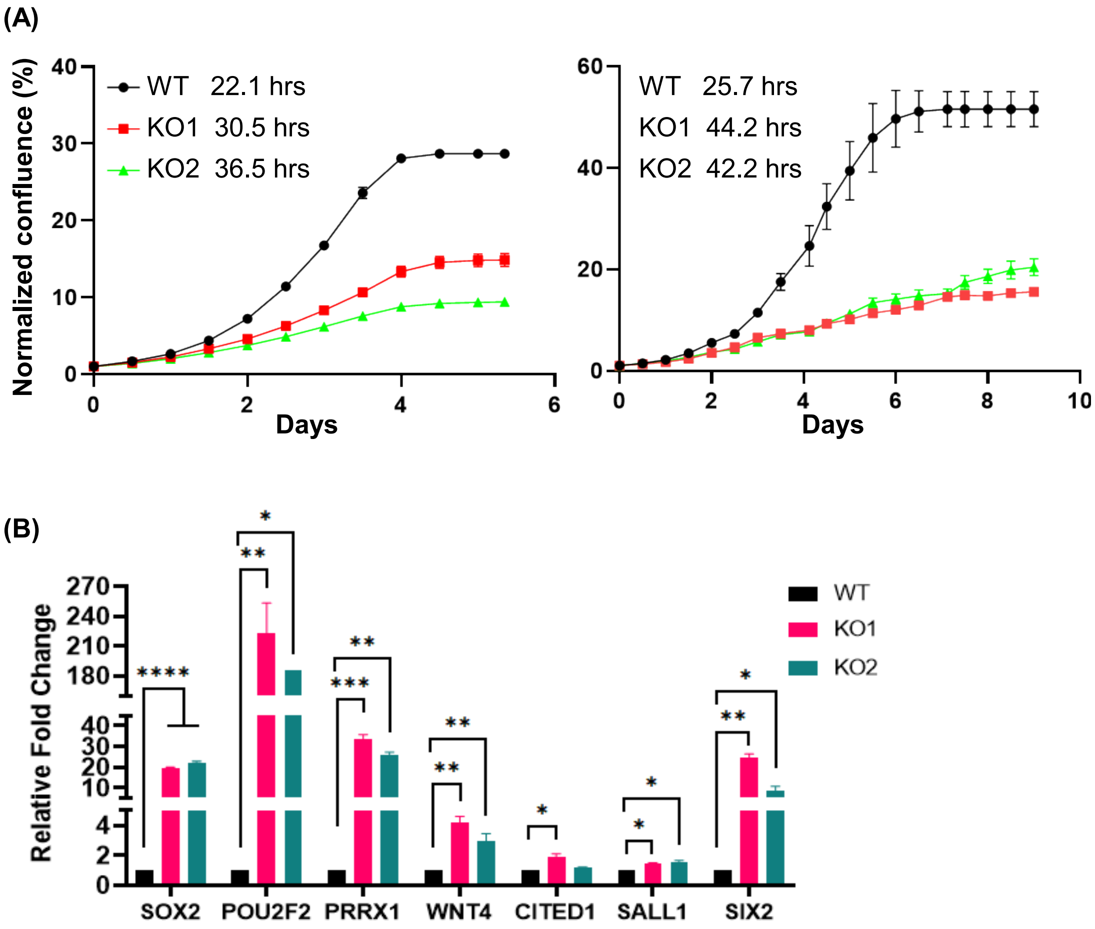

Supplemental Figure S4

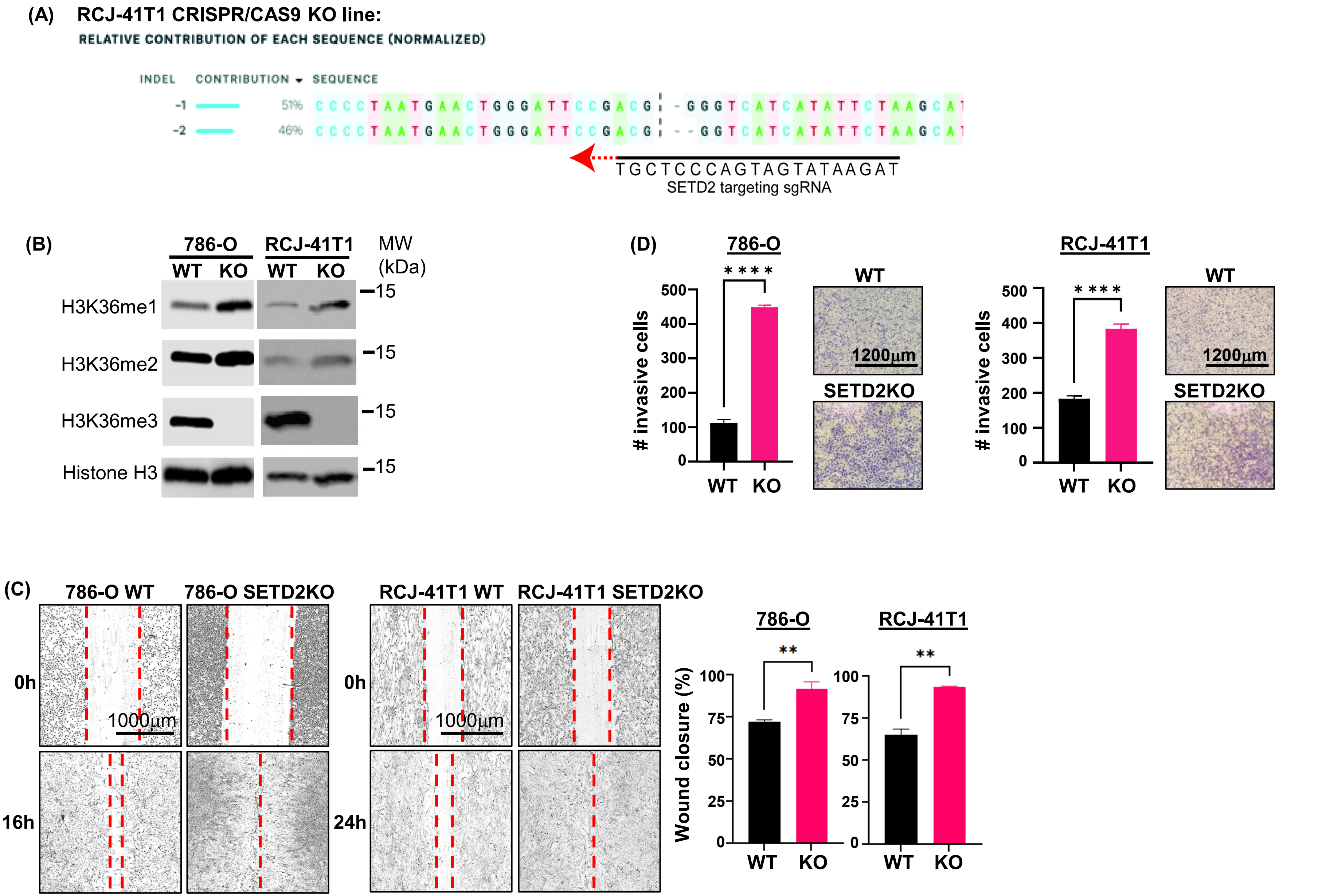

Supplemental Figure S5

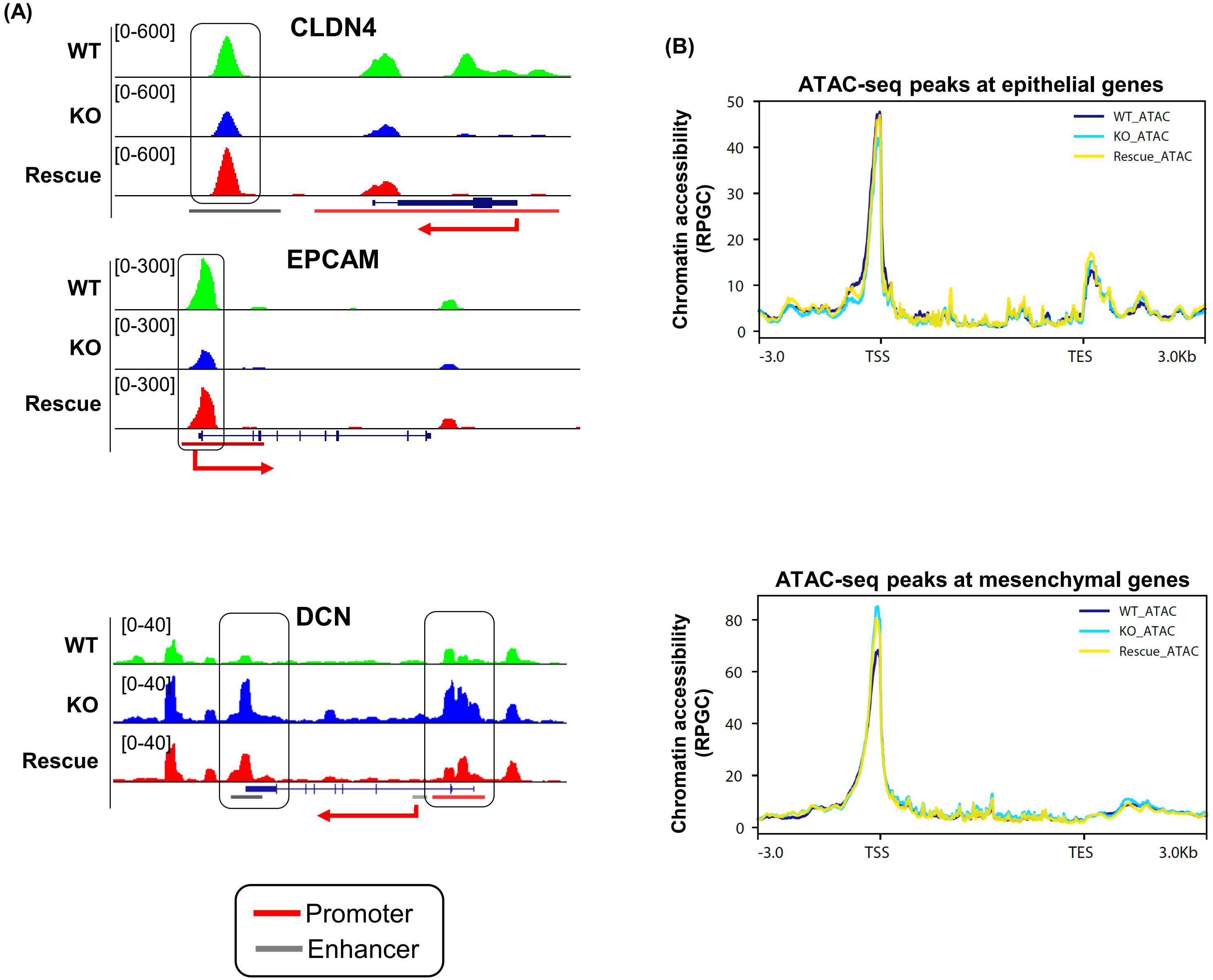

Supplemental Figure S6

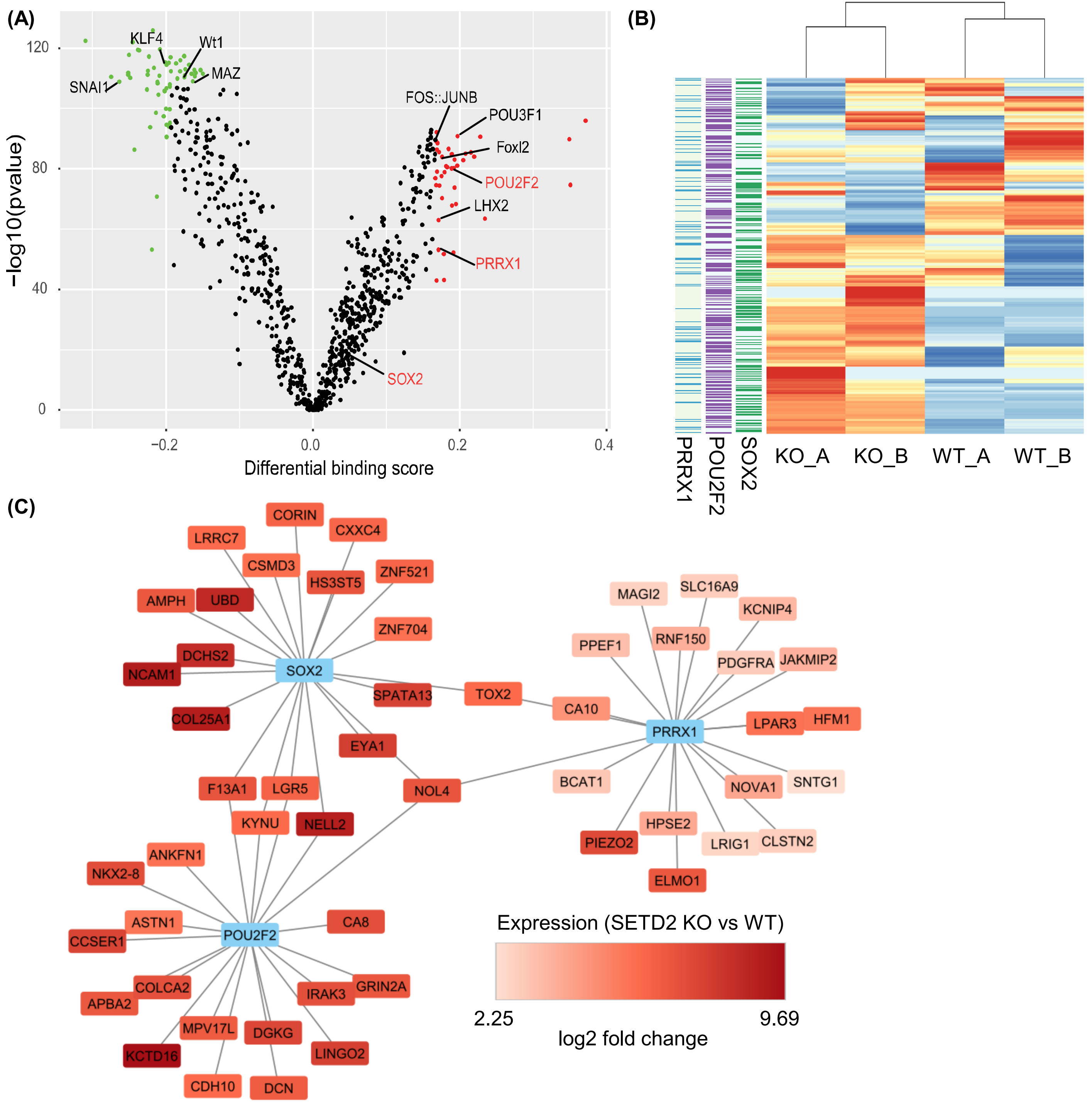

Supplemental Figure S7

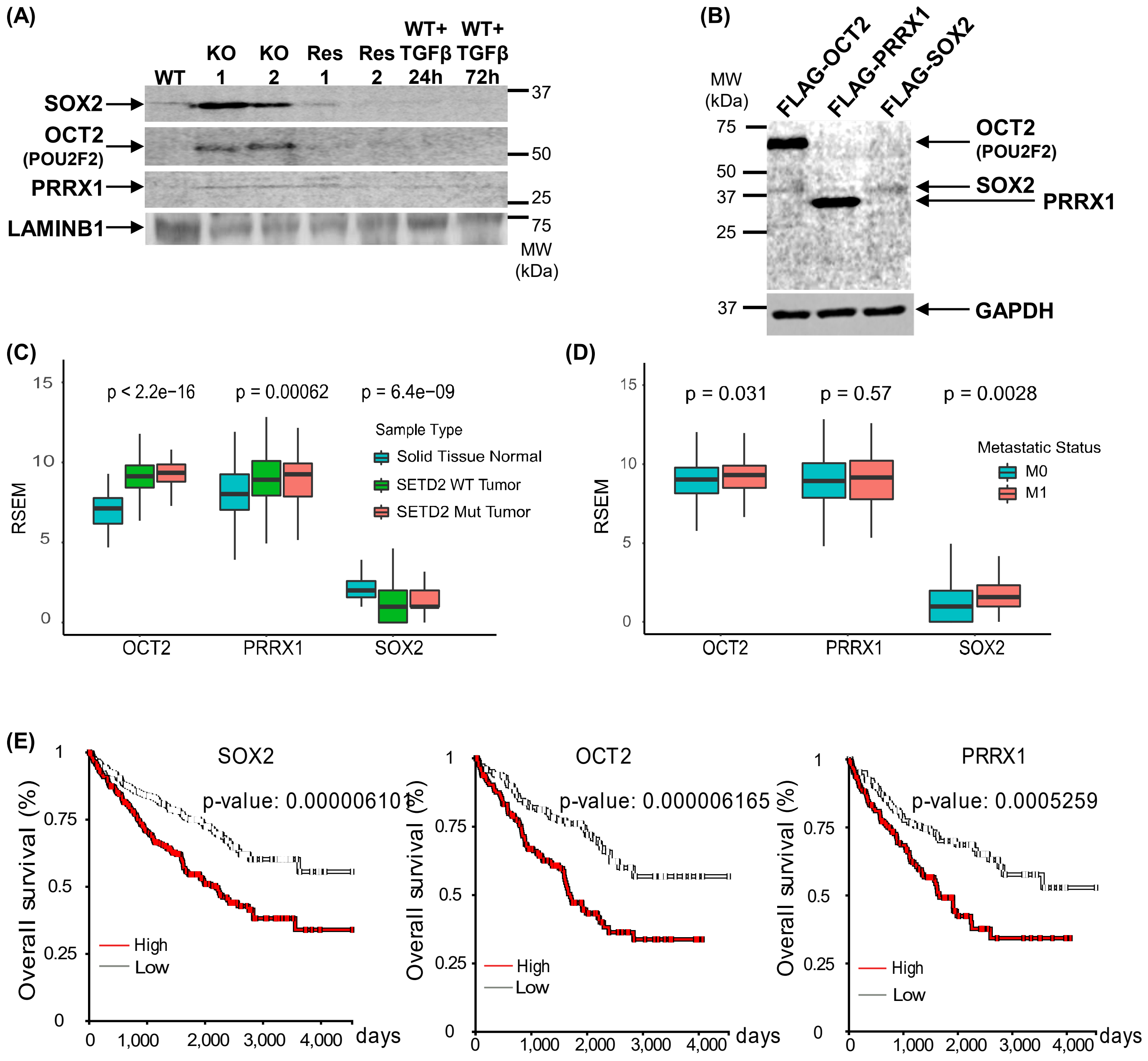

Supplemental Figure S8
